# Towards Tomography-Based Real-Time Control of Multiphase Flows: A Proof of Concept in Inline Fluid Separation

**DOI:** 10.3390/s22124443

**Published:** 2022-06-12

**Authors:** Matheus M. Garcia, Muhammad A. Sattar, Hanane Atmani, Dominique Legendre, Laurent Babout, Eckhard Schleicher, Uwe Hampel, Luis M. Portela

**Affiliations:** 1 Department of Chemical Engineering, Delft University of Technology, Van der Maasweg 9, 2629 HZ Delft, The Netherlands; l.portela@tudelft.nl; 2 Institute of Applied Computer Science, Lodz University of Technology, Stefanowskiego 18/22, 90-924 Lodz, Poland; muhammad.sattar@dokt.p.lodz.pl (M.A.S.); laurent.babout@p.lodz.pl (L.B.); 3 Institut de Mécanique des Fluides de Toulouse, Université de Toulouse, 2 Allée du Professeur Camille Soula, 31400 Toulouse, France; hanane.atmani@imft.fr (H.A.); dominique.legendre@imft.fr (D.L.); 4 Institute of Fluid Dynamics, Helmholtz-Zentrum Dresden-Rossendorf, Bautzner Landstraße 400, 01328 Dresden, Germany; e.schleicher@hzdr.de (E.S.); u.hampel@hzdr.de (U.H.); 5 Institute of Power Engineering, Technische Universität Dresden, 01062 Dresden, Germany

**Keywords:** tomography-based control, inline fluid separator, swirl separator, hydrocyclone, gas–liquid swirl flow, electrical resistance tomography, real-time control, multiphase flows

## Abstract

The performance of multiphase flow processes is often determined by the distribution of phases inside the equipment. However, controllers in the field are typically implemented based on flow variables, which are simpler to measure, but indirectly connected to performance (e.g., pressure). Tomography has been used in the study of the distribution of phases of multiphase flows for decades, but only recently, the temporal resolution of the technique was sufficient for real-time reconstructions of the flow. Due to the strong connection between the performance and distribution of phases, it is expected that the introduction of tomography to the real-time control of multiphase flows will lead to substantial improvements in the system performance in relation to the current controllers in the field. This paper uses a gas–liquid inline swirl separator to analyze the possibilities and limitations of tomography-based real-time control of multiphase flow processes. Experiments were performed in the separator using a wire-mesh sensor (WMS) and a high-speed camera to show that multiphase flows have two components in their dynamics: one intrinsic to its nonlinear physics, occurring independent of external process disturbances, and one due to process disturbances (e.g., changes in the flow rates of the installation). Moreover, it is shown that the intrinsic dynamics propagate from upstream to inside the separator and can be used in predictive and feedforward control strategies. In addition to the WMS experiments, a proportional–integral feedback controller based on electrical resistance tomography (ERT) was implemented in the separator, with successful results in relation to the control of the distribution of phases and impact on the performance of the process: the capture of gas was increased from 76% to 93% of the total gas with the tomography-based controller. The results obtained with the inline swirl separator are extended in the perspective of the tomography-based control of quasi-1D multiphase flows.

## 1. Introduction

Multiphase flows are found in a wide range of industrial processes, e.g., in heat exchangers [1,2], bubble column reactors [3], the oil and gas industry [4,5], and nuclear power plants [6,7]. A fundamental aspect of multiphase flows is that the distribution of fluids (phases) in the flow plays the major role in the performance and safety of the equipment. For instance: (i) the mass transfer in bubble column reactors is connected to the gas–liquid interfacial area [8,9]; (ii) the mixture is divided into single-phase streams in an ideal separation process; (iii) slug flows cause fluctuating forces in the oil pipelines, which can lead to environmental disasters [10,11]. Therefore, it is desirable to include the distribution of phases in the real-time control of multiphase flow processes.

Several techniques have been developed over the years to measure the distribution of fluids in multiphase flows. For instance, wire-mesh sensors (WMSs) have been used to measure the cross-sectional profiles of the volumetric fraction in gas–liquid [12,13], liquid-liquid [14,15] and three-phase [16,17] flows. In the field of industrial tomography, soft-field techniques such as electrical resistance [18,19], electrical capacitance [20,21], and electrical impedance tomography [22] have been applied in the study of the distribution of the phases and volumetric fractions of multiphase flows, and more sophisticated (hard-field) techniques such as MRI [23,24,25] and ultrasound [26,27] have been applied in the measurement of both the volumetric fraction and flow velocity. However, until recently, these techniques were still limited to slow processes or offline applications, and the real-time control of multiphase flows was limited to flow variables that are faster to measure, but are only indirectly connected to performance, such as pressure [28,29], density, or flow rate [30].

The main challenges in the implementation of real-time controllers based on the distribution of phases comes from: (i) the short time scales of the distribution of fluids in multiphase flow processes, and (ii) the temporal resolution of tomography.

From the flow side, it is shown later in Section 4 that the multiphase flow dynamics can be split from a control perspective into two components: (i) one intrinsic to the process, which is connected to the complex nonlinear physics of multiphase flows and occurs even for fixed nominal operating conditions of the system (e.g., flow rates and pressure), and (ii) one extrinsic to the process, occurring only when changes in the operating conditions of the equipment are present (process disturbances).

**Figure 1:**
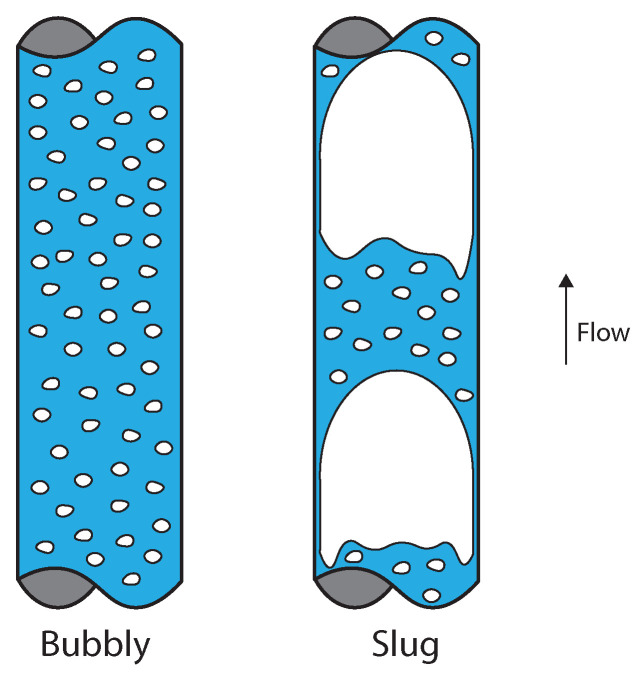
Distribution of gas (white) and liquid (blue) in vertical gas–liquid flows of the bubbly (**left**) and slug (**right**) flow regimes.

The intrinsic dynamics is strongly connected to the multiphase flow patterns. In vertical gas–liquid flows, the flow pattern depends on the flow rates of liquid and gas. For example, in Figure 1, two common patterns are shown: bubbly and slug.

The bubbly and slug flow patterns occur for (distinct) fixed flow rates of liquid and gas. The physics of the flow distribute the gas, continuously injected upstream in the pipe, into a stream of small bubbles (bubbly flow) or into a flow that contains large gas pockets (slug flow). If a sensor is installed in the pipe to monitor the distribution of phases, it will observe an intrinsically unsteady distribution of fluids. This intrinsic dynamics is different in the two cases and is independent of external (extrinsic) actions.

If changes in the boundary conditions of the flow are performed (e.g., an increase in the flow rate of one of the phases), additional transient effects are going to be observed by the sensor on top of the intrinsic dynamics, corresponding to the extrinsic dynamics.

The distinction between intrinsic and extrinsic dynamics is particularly interesting in terms of monitoring and control: while intrinsic dynamics dominate the frequencies in the order of Hertz (e.g., the frequency of slugs in a gas–liquid flow is typically above 1 Hz [31,32]), process disturbances are typically slow, in the order of minutes, and the extrinsic dynamics often reach a quasi-steady state. Therefore, the control of the system in relation to (external) process disturbances can be performed by relatively slow controllers, while the control of the intrinsic dynamics requires fast controllers.

From the tomography side, the main challenge comes from the obtainment of tomographic data, which is performed in two steps: data acquisition and image reconstruction. The data acquisition can be performed at relatively high frequencies [33]; however, the image reconstruction is typically computational demanding and, therefore, slow [34].

The fast acquisition of flow data is one of the reasons for the many successful applications of tomography in the study of multiphase flows mentioned at the beginning of the section, and the slow image reconstruction was the reason behind the limitation of tomography to offline or slow applications; the recent increase in computational power together with the development of optimized reconstruction algorithms finally allows acquiring and reconstructing tomographic data at speeds compatible with the intrinsic multiphase flow dynamics [35,36], opening the door to applications of the real-time control of the distribution of phases of multiphase flows, strongly connected to the equipment performance, using tomography.

This paper adopts a gas–liquid inline swirl separator (ISS), introduced in Section 2, to explore the possibilities and limitations of the tomography-based real-time control of multiphase flows. Two studies are performed: (i) the potential application of tomography (or similar techniques) in feedforward or predictive controllers, which is made based on the analysis of the connection between wire-mesh sensor measurements upstream of the separator, and camera recordings of the flow inside the separator (Section 4); (ii) the potential application of tomography in feedback controllers for the distribution of phases, which is performed via a proportional–integral controller, which acts on the system based on the measurement of the distribution of fluids (in particular, the size of the gas core) by electrical resistance tomography (Section 5). The results obtained with the inline swirl separator are extended to the field of quasi-1D multiphase flows in Section 6.

## 2. A Tomography-Controlled Inline Swirl Separator

**Figure 2:**
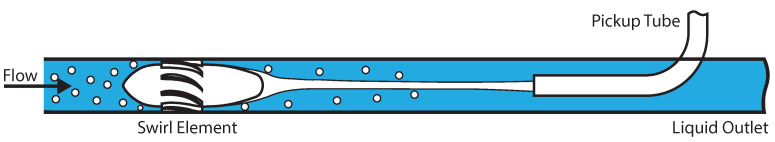
Gas–liquid inline swirl separator. The gas–liquid mixture of the upcoming flow is split into a continuous core of gas and a surrounding annulus of liquid due to the swirl. The separation is performed by the pickup tube, which collects the flow in the center of the separator.

A gas–liquid inline swirl separator (ISS) was chosen as representative of the multiphase flow field in this paper due to its industrial relevance, since separation technologies are fundamental to many branches of the process industry: oil and gas [37], nuclear power plants [38], wastewater treatment [39], etc. Moreover, the separator represents a wide range of multiphase processes, since it covers both the typical behavior of a mixture (while the phases are still mixed in the beginning of the separator) and the strong change in the distribution of fluids that occurs in some processes (during the separation). The schematic of a gas–liquid inline swirl separator is presented in Figure 2.

Inline swirl separators rely on large centrifugal accelerations (in the order of 100 g) to separate a mixture of fluids of different densities [40]. The flow starts to swirl when the mixture of fluids crosses the swirl element, leading to centripetal forces, which push the lighter of the two fluids (gas) to the center of the pipe, where it coalesces into a continuous core. The system is designed to operate in conditions in which the gas core formed inside the separator is roughly centered and axisymmetric, being successfully captured by the pickup tube with most of the liquid proceeding to the liquid outlet.

Since there are no additional inlets in the ISS, apart from the pipe reaching the swirl element, it is expected that the gas core inside the inline swirl separator is connected to a great extent to the flow upstream of the swirl element, for both extrinsic disturbances and intrinsic dynamics; the connection is investigated using a double-layer wire-mesh sensor (WMS) installed upstream of the swirl element.

**Figure 3:**
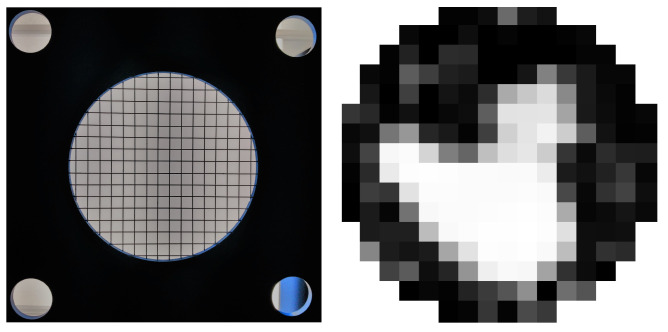
(**Left**) Picture of the wires of one wire-mesh sensor. The two planes of parallel wires are installed perpendicular to each other, creating a virtual grid of points; (**Right**) Volume fraction of gas measured by the sensor, with the gas fraction proportional to the grayscale of the pixel (pure liquid is black, and pure gas is white).

The wire-mesh sensor consists of two planes of parallel wires (transmitter and receiver) installed separated by a small distance and perpendicular to each other, forming a grid of virtual intersections between the wires. A picture of one wire-mesh is presented in the left image of Figure 3.

The conductance of each virtual point in the grid of the wire-mesh sensor is measured by creating an electric potential between one transmitter wire and all the receiver wires, which have their electric currents measured. This leads to the measurement of conductance along one line of the sensor (defined by the transmitter wire); once all the wires of the transmitter plane are covered, a 2D profile of conductance is obtained. The conductance distribution is converted to the distribution of phases assuming a proportional relation between conductance and the volumetric fraction of the liquid ($\varepsilon_{liq}$) in each point (i,j) of the grid [41]:

(1) $$\varepsilon_{liq}\left(i,j\right)=\frac{G(i,j)}{G_{liq}\left(i,j\right)},$$

where G(i,j) is the conductance being measured at each point and $G_{liq}\left(i,j\right)$ is the conductance measured at each intersection with only liquid in the domain. An example of the volumetric fraction profile obtained by the WMS is shown in the right image of Figure 3.

The double-layer wire-mesh sensor consists of two individual WMS installed separated by a small distance, usually in the order of cm. In this configuration, the gas velocity can be estimated by cross-correlating in time the liquid fraction profiles measured by each wire-mesh sensor; the gas velocity is calculated dividing the distance between the wire-mesh sensors by the shift in time that maximizes the cross-correlation between their measurements.

**Figure 4:**
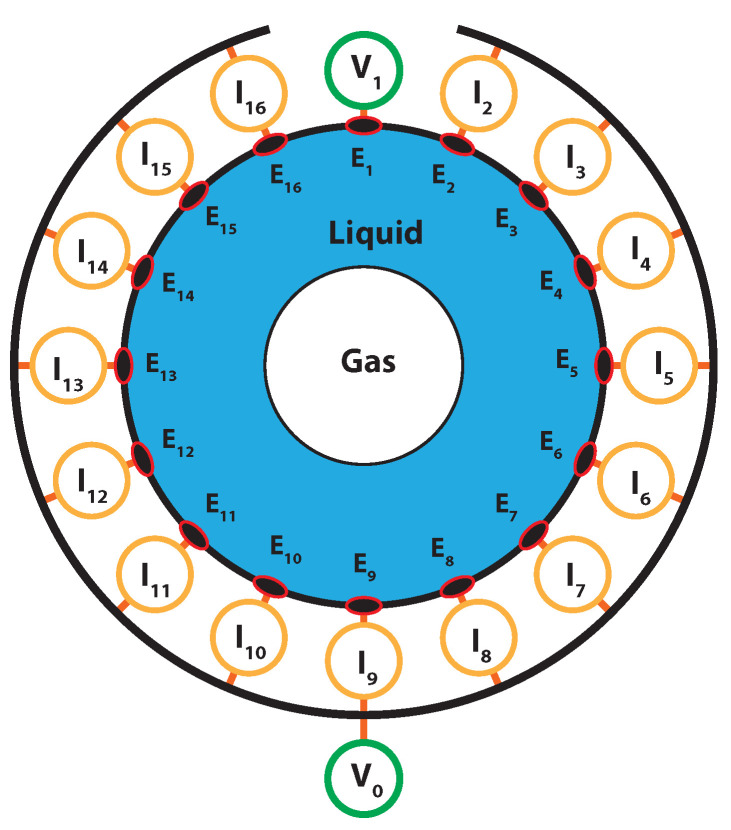
Electrical resistance tomography with 16 electrodes ($E_{1}$ to $E_{16}$) installed in the inline swirl separator. In this image, electrode 1 is used as the source ($V_{1}$), and the remaining electrodes act as the sink. The sink electrodes are kept grounded at $V_{0}$ and have their electric currents measured (*I*). Image adapted from [40].

In addition to the analysis performed with the wire-mesh sensor, an electrical resistance tomography (ERT) sensor is installed in the separator to provide the feedback of the distribution of phases in the process for real-time control applications. The technique is used to monitor the gas core near the pickup tube, a natural choice since its size and position, in relation to the size and position of the pickup tube, determine the capture of liquid and gas by each outlet of the separator. The ERT sensor reconstructs the distribution of phases in the flow based on electrical measurements (voltage and current) in electrodes placed in the wall of the equipment, as shown in Figure 4.

The ERT acquisition system used in the experiments creates a voltage in a source electrode and measures the currents generated in the remaining electrodes, which are kept grounded and act as the sink. The data required to reconstruct one frame of the flow are obtained when the current measurements are performed for each electrode acting as the voltage source, leading to a total of N(N−1) measurements, where *N* is the total number of electrodes in the acquisition system.

**Figure 5:**
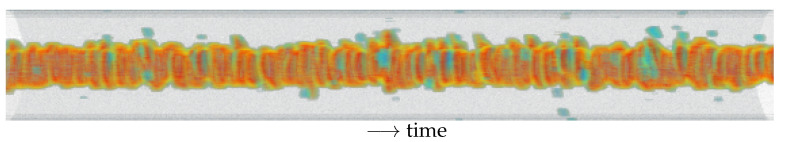
Gas core inside the inline swirl separator measured and reconstructed using classical ERT. A 2D sensor is used in the measurements, and the 3rd axis of the image (axial direction) corresponds to time.

The current–voltage relation measured in the electrodes depends on the distribution of electrical conductivity in the flow, which must be reconstructed from the boundary measurements. In classical electrical resistance tomography, this is usually performed via an inverse problem based on the Maxwell equations, with the domain discretized using the finite element method. An image of the distribution of fluids measured and reconstructed by classical ERT algorithms is presented in Figure 5.

The solution of the inverse problem in ERT is typically time consuming and requires iterative schemes to achieve a satisfactory accuracy in the reconstructed image. Therefore, even if the electrical measurements can be performed at high frequencies, the reconstruction step limits the online measurement of the distribution of phases in the flow to low temporal resolutions.

In order to speed-up the ERT measurements, which is fundamental to control applications, the inline swirl separator application-specific reconstruction algorithm of [36] is used in this work. The algorithm, which is detailed in Section 3.4, reconstructs the gas core size and position directly from the current measurements using equations that are simple and fast to compute, providing a feedback of the separation much faster than applications based on the inverse problem. Despite the measurement of both the gas core size and position, only the core size detected by the ERT is forwarded to the feedback controller, since the gas core is roughly in the center of the pipe.

**Figure 6:**
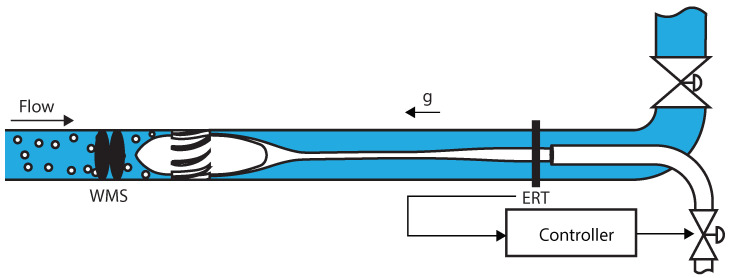
Inline swirl separator with wire-mesh and electrical resistance tomography sensors. The feedback controller of the image connects the gas core size, measured by ERT, to inputs in the pickup tube valve.

The ERT-based feedback controller uses a control valve installed in the pickup tube to act on the separation. The controller is designed to reject external disturbances in the process (in particular, changes in the gas flow rate of the installation) and connects the gas core size measured by ERT (system output) to actions in the pickup tube valve (system input), as shown in Figure 6.

## 3. Experimental Setup

This section describes the flow loop, wire-mesh sensor, high-speed camera, and ERT system used in the experiments.

### 3.1. Flow Loop Facility

**Figure 7:**
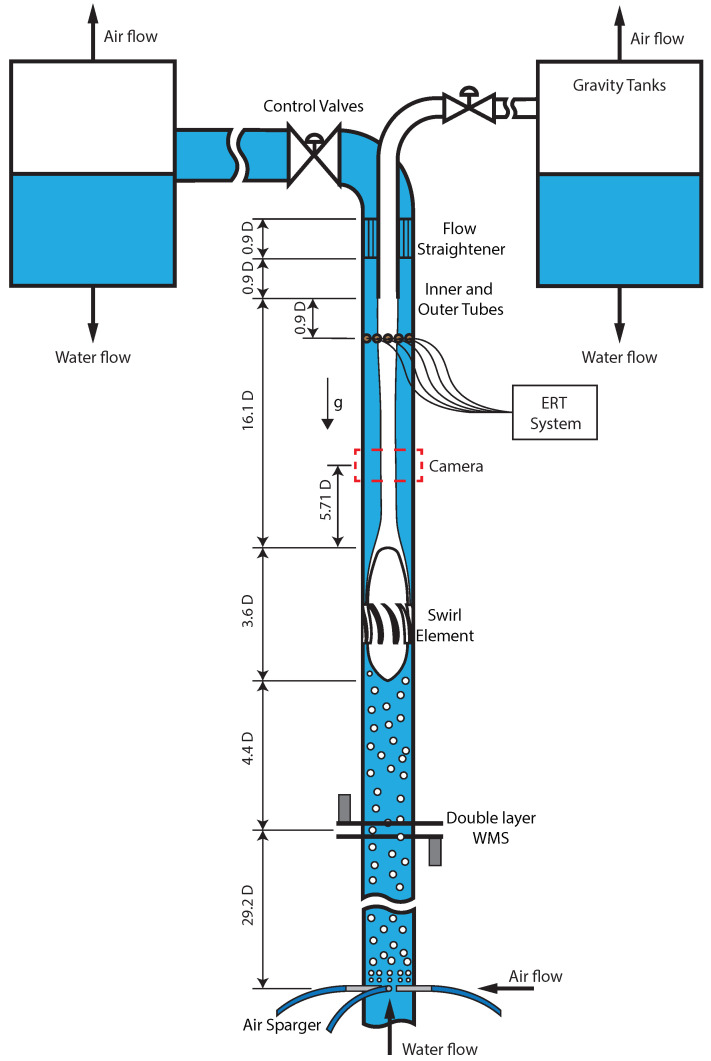
Main section of the Inline Swirl Separator of the Delft University of Technology. The separator has an inner diameter D=81.4 mm.

The Inline Swirl Separator Facility of the Delft University of Technology was used in the experiments of this paper. It consists of a vertical separator with an inner diameter D=81.4 mm and runs with a mixture of air and water. A schematic of the Facility with its most relevant dimensions is presented in Figure 7.

A centrifugal pump is used to generate the water flow rate in the flow loop, which is measured by a Krohne Optiflux 2100 C water magnetic flow meter; the flow meter measures water flow rates up to 350 L/min with accuracy ±(0.12 L/min + 0.3% mv), where mv corresponds to the measured value (the uncertainty depends on the water flow rate being detected by the sensor). The air flow rate in the system is imposed by a Bronkhorst El-Flow Select Mass Flow Controller, with a nominal range between 20 and 1000 Ln/min and accuracy ±(1 Ln/min + 0.5% mv). The normal liters per minute (Ln/min) in the sensor output is calculated from the measured mass flow rate considering the density of air at 1 atm and 0 °C. A vertical pipeline of length 33.6D is present between the creation of the air–water mixture and the swirl element, allowing the two-phase flow to develop considerably before reaching the separator.

The separator starts at the swirl element, which has a design swirl number [42] of Ω=4. The swirl tube, where the centrifugal effects separate the flow into a core of air and an annulus of water, is 16.1D long, ending at the tip of the pickup tube. The pickup tube, responsible for capturing the air in the flow, is made of PVC and has an inner diameter of 36 mm (0.44D) and an outer diameter of 40 mm (0.49D).

The pickup tube splits the flow into two streams, which proceed to individual gravity tanks. In the gravity tank connected to the pickup tube, the water flow rate of the stream is measured by an Endress+Hauser Promag 10D magnetic flow meter with accuracy ±(0.06 L/min + 0.5% mv), and the air flow rate is measured by a Bronkhorst Mass-Stream Flow Meter with accuracy ±(5 Ln/min + 0.5% mv). In the gravity tank connected to the liquid outlet, the water flow rate is measured by a Krohne Optiflux 2100 with accuracy ±(0.12 L/min + 0.3% mv) and the air flow rate by a Bronkhorst Mass-Stream Flow Meter with accuracy ±(1 Ln/min + 0.5% mv).

The efficiency of the system in relation to the capture of air by the pickup tube and water by the water outlet is obtained comparing the flow rates measured in the gravity tanks with the total flow rates of each phase in the system. The water efficiency is defined as the ratio between the water flow rate in the liquid outlet ($q_{w,lo}$) and the total water flow rate of the loop ($q_{w,in}$):

(2) $$\eta_{w}≜\frac{q_{w,lo}}{q_{w,in}},$$

and the air efficiency is defined as the ratio between the air mass flow rate captured by the pickup tube (${\dot{m}}_{a,pt}$) and the total air mass flow rate of the system (${\dot{m}}_{a,in}$):

(3) $$\eta_{a}≜\frac{{\dot{m}}_{a,pt}}{{\dot{m}}_{a,in}}$$

The uncertainty in the calculation of the water efficiency can be improved if the measurements of the two gravity tanks are combined according to

(4) $$\eta_{w}=\frac{1}{2}\left(\frac{q_{w,lo}}{q_{w,in}}+\frac{q_{w,in}-q_{w,pt}}{q_{w,in}}\right),$$

since the flow rate of water in the liquid outlet is equal to the injection flow rate minus the pickup tube flow rate from the conservation of mass.

Since the accuracy of the air flow meter connected to the water outlet (1 Ln/min + 0.5% mv) is much better than the accuracy of the air flow meter connected to the pickup tube (5 Ln/min + 0.5% mv), a greater precision in the air efficiency is achieved if only the liquid outlet meter is used:

(5) $$\eta_{a}=\frac{{\dot{m}}_{a,in}-{\dot{m}}_{a,lo}}{{\dot{m}}_{a,in}},$$

and the pickup tube meter is used in the experiments to check the consistency of the air mass flow rates measured in the liquid outlet.

The relative importance between the water and air efficiencies is application-dependent. For instance, if removing all the gas from the liquid is crucial to the process, then the controller should be designed targeting a unitary air efficiency. Analogously, if the application requires pure gas in the pickup tube, then the controller should target a water efficiency equal to one. Since there is no particular preference for either air or water in the study of this paper, the average between the two efficiencies is used to summarize the separation into a single variable:

(6) $$\eta_{m}=0.5\left(\eta_{w}+\eta_{a}\right)$$

For the flow rates used in the experiments, the uncertainties of the air, water, and mean efficiencies are estimated from the flow meter uncertainties as $\delta (\eta_{a})=\pm 0.8\%$, $\delta (\eta_{w})=\pm 0.2\%$, and $\delta (\eta_{m})=\pm 0.4\%$, respectively.

### 3.2. The Double-Layer Wire-Mesh Sensor

**Figure 8:**
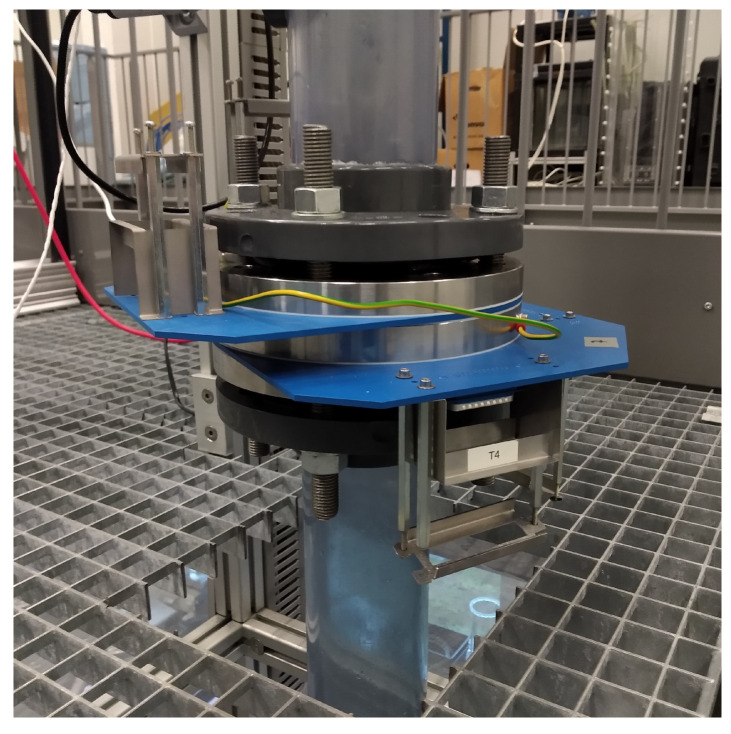
Double-layer wire-mesh sensor installed in the Flow Facility of the Delft University of Technology.

A double-layer wire-mesh sensor (WMS) is used in the investigation of the connection between the gas core inside the inline swirl separator, which is measured by a high-speed camera, and the flow upstream of the swirl element, which is measured by the WMS. The double-layer WMS is installed 4.4D upstream of the swirl element, as previously shown in Figure 7. A picture of the device installed in the flow loop is presented in Figure 8.

Each WMS sensor of the double-layer configuration has a mesh of 16 × 16 wires and measures the flow at 5 kHz. During the cross-correlations with the camera reported in Section 4.3, the wire-mesh signal is averaged every four frames to match the camera frequency (1.25 kHz); during tests, a similar cross-correlation was obtained if only one every four frames is considered in the WMS data, mimicking an acquisition at 1.25 kHz.

The comparisons with the camera of Section 4.3, including the cross-correlation, were made based on the time series of the gas fraction (void fraction) averaged across the cross-section of the pipe at the wire-mesh sensor location, which is calculated from the 2D distribution of liquid and gas measured by the sensor for each frame according to

(7) $$\alpha_{wms}\left(t\right)=1-\frac{1}{\pi R^{2}}\int_{0}^{2\pi }\int_{0}^{R}\varepsilon_{liq}\left(r,\theta ,t\right)rdrd\theta$$

In addition to the distribution of phases upstream of the separator, the average gas velocity at the location of the double-layer wire-mesh sensor can be estimated by cross-correlating the two time series of the spatially averaged void fraction ($\alpha_{wms}$) obtained by each WMS in the double layer and diving the distance between the two layers (21.9 ± 0.1 mm) plus the width of one layer (3.8 mm) by the time-shift that maximizes the correlation between the signals.

Three-dimensional flow images can be made by plotting the cross-sectional measurement of the wire-mesh sensor (e.g., Figure 3, right) against time, with the resulting figure resembling a volumetric reconstruction of the distribution of phases in the flow. However, there is a fundamental difference between the image obtained by stacking cross-sectional measurements over time and the real 3D distribution of fluids in space: since the distribution of phases changes as the flow is transported along the pipe towards the wire-mesh location, the distribution of fluids detected by the WMS is always different from the distribution of fluids observed in upstream locations at previous instants of time, and the time axis of the image cannot be perfectly converted into space using the gas velocity.

The reconstruction of the wire-mesh sensor data into 3D images is particularly interesting when analyzing the multiphase flow patterns and their intrinsic dynamics. For instance, later in this paper (Figures 12 and 13), the distribution of fluids measured along a single wire of the WMS (one of the central wires of Figure 3, left) is plotted against time and (i) presented together with the time series of the spatially averaged void fraction, clearly showing the connection between the two quantities, or (ii) compared to recordings of the gas core reconstructed in a similar way (described in Section 3.3), resulting in a visual confirmation of the link between the intrinsic dynamics of the upstream flow and of the gas core (Figure 19).

### 3.3. High-Speed Camera

A high-speed camera (Basler acA1920 150uc with 8 mm lenses by Computar) was used to measure the gas core inside the inline swirl separator in the experiments of Section 4. The camera is controlled by LabVIEW and records the gas core at 1250 frames per second with a spatial resolution of 3.4 pixels/mm. Since the cross-section of the gas core is roughly circular at each position along the pipe axis, a single camera view is required to extract the core size as a function of the axial position from the flow images.

**Figure 9:**
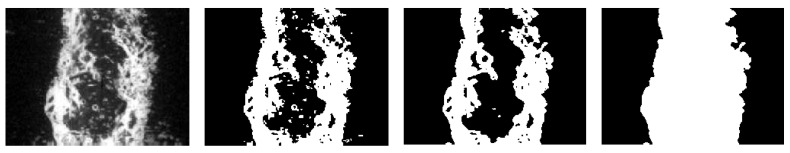
Image processing steps performed by the MATLAB code. From left to right: raw image obtained by the high-speed camera, binary image, image after the moving average filter, and final image with holes filled.

The gas core size is recovered from the camera recordings following the image processing steps of Figure 9, which were implemented in MATLAB. First, the grayscale images of the camera are made binary based on a threshold calculated by the Otsu method [43] using the imbinarize function of the software. Then, a moving average of the pixel values (0 for black and 1 for white) is used to remove small white pixels from the image, corresponding to small bubbles or noise; the moving average sets the center pixel of a (moving) 5 × 5 window (25 pixels) to black if the average pixel value inside the window is below 0.5. Finally, the holes of the image are filled using the imfill function of MATLAB.

As the pipe of the inline separator has a cylindrical wall, the gas core observed in the images obtained by the camera is different from the core gas core inside the separator due to refraction. The effect is compensated by correcting the size of the pixels in the images based on Snell’s law, according to the strategy described in [35].

Once the size of each pixel is corrected to inside the pipe, the diameter of the gas core as a function of the axial position in the pipe for each snapshot is obtained by summing the width of all white pixels along the width of the core (horizontal direction in Figure 9). The result is then averaged along the length of the core (vertical direction in Figure 9) between 5.69D and 5.73D downstream of the swirl element; the length of 0.04D in the axial direction of the pipe was chosen to match the width of one layer of the double-layer WMS. The result obtained was interpreted as the core size in the camera location at the time of the picture and was converted to mm and normalized by the pipe diameter (81.4 mm).

The analysis of Section 4 was performed based on the time series of the gas volume fraction detected by the camera, which is calculated from the normalized core size as

(8) $$\alpha_{cam}\left(t\right)=d^{2}\left(t\right),$$

where d(t) corresponds to the normalized core size, previously obtained by averaging the gas core of each frame in the camera recordings over the 0.04D-long window.

By analogy to the plots made with the WMS, in which 2D data is plotted against time (described in Section 3.2), the gas core observed in the 0.04D-long window of the camera images can be approximated as the measurement of the gas–liquid distribution along a line perpendicular to the pipe axis and plotted against time to generate a 2D image of the flow (e.g., in Figures 14, 15 and 19 presented later in the paper). Please note that, despite the visual similarity, the resulting image is different from a flow picture since it has time and not space (the direction along the centerline of the pipe) as the second dimension.

### 3.4. The Real-Time Electrical Resistance Tomography Sensor

**Figure 10:**
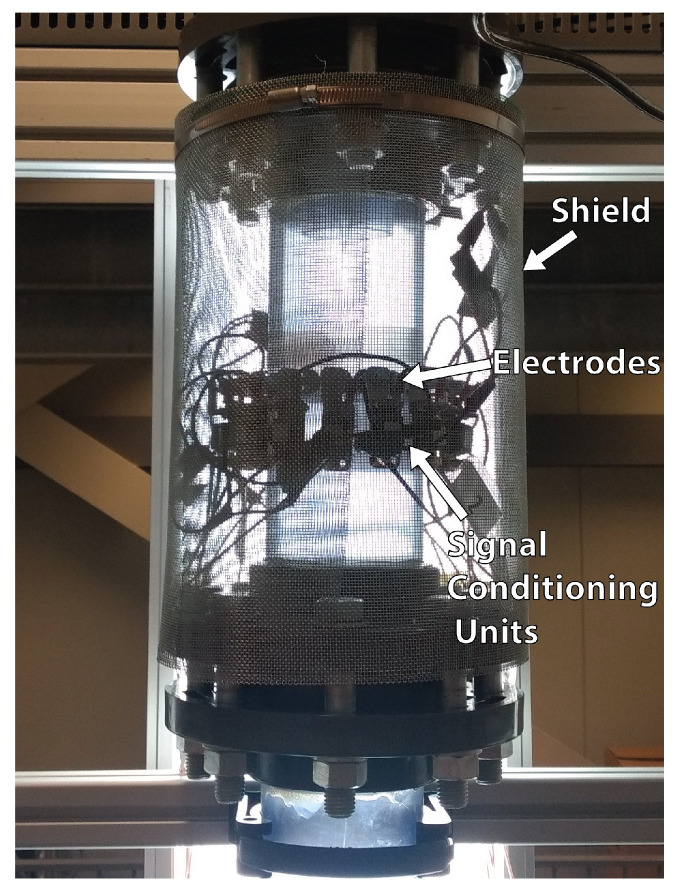
Electrical resistance tomography sensor installed in the pipe of the inline swirl separator. The electrodes and signal conditioning units can be seen inside the shield, used to isolate the measurement region from external electromagnetic effects.

An electrical resistance tomography (ERT) system with 16 electrodes was used to control the separation in Section 5. The electrodes of the sensor are M5 screws with a 12 mm button head, installed spaced 4mm in a single plane 0.9D below the pickup tube, as shown in Figure 7. A picture of the ERT system installed in the pipe of the separator with a shield to reduce the effects of external electromagnetic disturbances in the measurements is shown in Figure 10.

The Flow Watch ERT system from Rocsole Ltd was able to provide reliable measurements of gas–liquid swirling flows in previous works [35,36] and was used in the experiments of this paper. It operates in the voltage–current (VC) scheme, i.e., the measurements were performed imposing a voltage in one electrode (source), while measuring the current in the others, which were kept grounded (sink), and a frame of the flow was obtained when every electrode of the system acted as the source (Section 2). For the 16-electrode system used, a frame of the flow was obtained when 16×15=240 current measurements were performed.

The Flow Watch ERT system is able to measure the flow at a maximum frequency of 12 frames per second (1 frame = 240 currents) and operated at 10 frames per second in the experiments of Section 5.

If classical ERT were used to measure the gas core inside the separator, the cross-sectional profile of the electric conductivity of each frame would be reconstructed from the 240 current measurements solving the inverse problem, and the gas core would be extracted from the image based on the region of low conductivity [35]. However, it is known that the gas core inside the ISS is a single element of a roughly circular cross-section. Therefore, an application-specific algorithm can be written to search for the size (*d*) and centroid position (r,θ) of the electrically insulating gas core directly from the current measurements, skipping the reconstruction of the conductivity profile by the inverse problem. As consequence, the measurement of the gas core is simplified from a complex inverse problem to a system of three equations and three variables, which can be solved almost instantaneously.

The application-specific ERT reconstruction algorithm of [36] was used to reconstruct in real-time the gas core inside the inline fluid separator based on the raw data of current measured by the acquisition system. The algorithm is based on the voltage (V)–current (I) measurements of opposite electrodes (following Figure 4, electrodes 1 (V) and 9 (I), 2 (V), and 10 (I), 3 (V) and 11 (I), ...or, in general, *m* (V) and m+8 (I)), which are normalized by the currents obtained with only water in the system, $i_{m,m+8}{|}_{water}$:

(9) $$\Delta i_{m,m+8}=1-\frac{i_{m,m+8}}{i_{m,m+8}{|}_{water}}$$

The gas core is electrically insulating and, therefore, increases the equivalent resistance between the electrodes of the system when present in the domain. As a consequence, since the voltage imposed by the Rocsole ERT system in the source electrode is always the same, the gas core causes a drop in the currents measured in the sink electrodes, i.e., $i_{m,m+8}\leq i_{m,m+8,water}$.

The decay in the current measured in the sink electrodes is dependent on the size and position of the gas core. Larger gas cores cause larger distortions in the electric field and, therefore, result in smaller electrode currents, and electrodes closer to the core are more affected by it. Therefore, the gas core size and position can be recovered directly from the eight opposite measurements of $\Delta i_{m,m+8}$.

Based on calibrations performed with ABS phantoms, the gas core size normalized by the pipe diameter is obtained from $\Delta i_{m,m+8}$ as

(10) $$d={\langle \Delta i\rangle }^{0.6095},$$

where $\langle \Delta i\rangle$ corresponds to the common average between the eight values of $\Delta i_{m,m+8}$.

The radial position of the centroid of the gas core, *r*, is linked to the standard deviation of $\Delta i_{m,m+8}$, and the angular position of the gas core, θ, is obtained analyzing $\Delta i_{m,m+8}$ together with four additional 90° current measurements, obtained between electrodes 1 and 5, 5 and 9, 9 and 13, and 13 and 1. Since the gas core is well centered in the conditions explored in Section 5, only the core size (*d*) is used in this paper, and the reader is referred to [36] for details regarding the calculation of the position of the core.

The reconstruction of the gas core size using Equation (10) was performed by a computer connected to the ERT acquisition system, and the User Datagram Protocol (UDP) was used to forward the result to the computer that controls the pickup tube valve.

### 3.5. Actuators and Control

**Figure 11:**
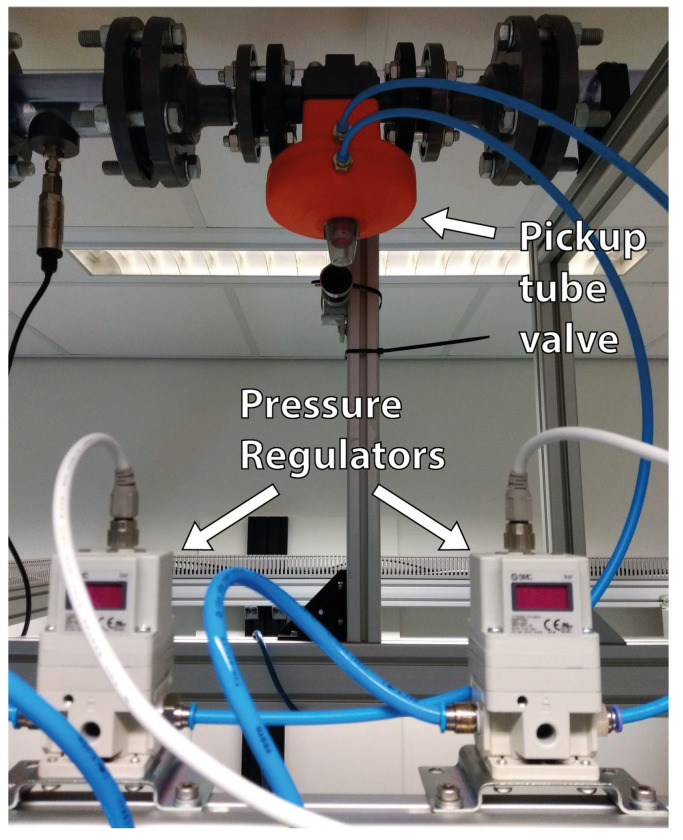
Pickup tube valve (orange) with its two pressure regulators (light gray).

The feedback controller of Section 5 uses the core size measured by ERT to compute control actions in the pickup tube valve. The pickup tube valve is a DN25 ASF Stübe MV 310 normally open diaphragm valve, and its opening is adjusted by two SMC ITV2050 pressure regulators. Each pressure regulator is installed in one side of the cavity of the valve, and the equipment operates between 4 and 5barg. The valve is always fully open at 4barg and fully closed at 5barg, but the exact values in which the extremes in the valve position are reached vary greatly depending on the flow pressure and previous pressure inputs, due to hysteresis. A picture of the valve connected to the pickup tube with the two pressure regulators is presented in Figure 11.

A code was written in LabVIEW to receive the gas core size measured by ERT via UDP (system output), compute, based on the control law, the cavity pressure setpoint of one of the pressure regulators connected to the pickup tube valve (system input), and send the result to the equipment at a frequency of 10 Hz.

## 4. Multiphase Flow Dynamics

As in most multiphase flow applications, the gas core inside the inline swirl separator has two dynamic components: one intrinsic to the process, connected to the gas–liquid flow patterns upstream and inside the equipment, and one extrinsic to the process, caused by external disturbances in the operating conditions of the system (e.g., a change in the liquid or gas flow rate).

This section analyzes the intrinsic dynamics of the gas–liquid flow patterns upstream (Section 4.1) and inside the inline swirl separator (Section 4.2), together with the connection between the two locations, both in terms of external disturbances and intrinsic dynamics (Section 4.3). The study was performed based on the data obtained with the wire-mesh sensor (gas distribution in the upstream flow) and camera (gas core) for thirty-five different combinations of air and water flow rates, listed in Appendix A.

### 4.1. Vertical Non-Swirling Gas–Liquid Flow Patterns

Vertical gas–liquid flows without swirl can be split into four main patterns: bubbly, slug, churn, and annular [44]. However, only bubbly and slug flows were observed in the experiments in this paper.

**Figure 12:**
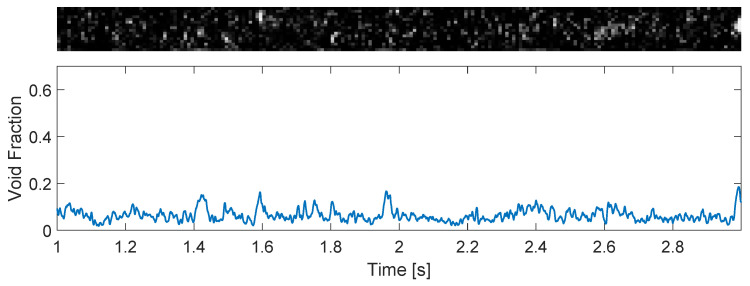
Gas–liquid distribution (**top**) and volume fraction of air (**bottom**) of a bubbly flow measured by the wire-mesh sensor installed upstream of the ISS for a water flow rate of 150 L/min and an air flow rate of 20 Ln/min. The two images correspond to the same data and share the same time axis. In the top image, white corresponds to gas and black to liquid, with the light intensity of gray pixels proportional to the local gas fraction measured by the WMS, such that bubbles smaller than the sensing area of the wire-mesh sensor are represented in gray.

Bubbly flows take place at relatively low flow rates of gas, leading to low gas volume fractions (void fractions) in the pipe and, as consequence, a stable distribution of gas into small bubbles. The distribution of phases detected by the wire-mesh sensor for one experimental point of Appendix A in the bubbly regime is shown in Figure 12, top, and the gas volume fraction obtained from the measurement according to Equation (7) is shown in Figure 12, bottom.

**Figure 13:**
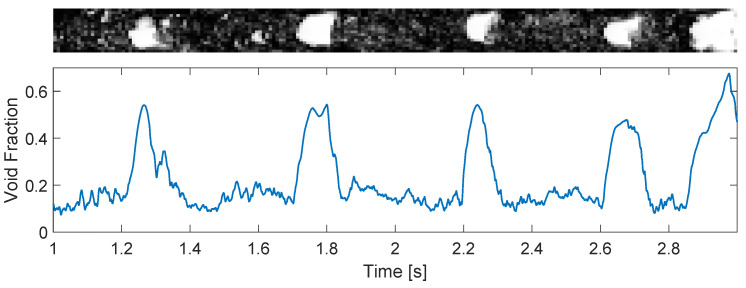
Gas–liquid distribution (**top**) and volumetric fraction of air (**bottom**) of a slug flow measured by the wire-mesh sensor installed upstream of the ISS for a water flow rate of 80 L/min and an air flow rate of 70 Ln/min. The two images correspond to the same data and share the same time axis. Gas is represented in white and liquid in black in the images, with the light intensity of gray pixels proportional to the local gas fraction measured by the WMS.

If the gas flow rate of the system is substantially increased, such that the volume fraction of air in the pipe is too large to maintain the separation of the bubbles, coalescence takes place and large pockets of air are formed in the pipe, leading to slug flow. Figure 13, top, shows the distribution of gas and liquid for one experimental point of Appendix A in the slug flow regime, and Figure 13, bottom, shows the gas volume fraction obtained for the condition using Equation (7). Figure 13 clearly shows the increase in the void fraction caused by the passage of large gas pockets across the wire-mesh sensor and the maintenance of a gas fraction of around 0.15 between the large gas pockets due to a continuous stream of small bubbles.

When comparing the bubbly and slug time series of the void fraction (top images of Figure 12 and Figure 13, respectively), it is clear that the oscillations in the void fraction have very different magnitudes depending on the flow pattern of the system. While the oscillations in the void fraction of bubbly flows (Figure 12) can be approximated as noise and neglected in practice, the intrinsic dynamics of slug flows (Figure 13) lead to the formation of large gas pockets in the flow and large-amplitude oscillations in the void fraction measured by the wire-mesh sensor; the intrinsic dynamics of slug flows cannot be neglected when designing process controllers targeting the rejection of external disturbances based on void fraction measurements.

### 4.2. Swirling Gas–Liquid Flow Patterns

When swirl is added to vertical gas–liquid flows, two additional flow patterns are observed in relation to the non-swirling case: stable and intermittent column [45].

**Figure 14:**
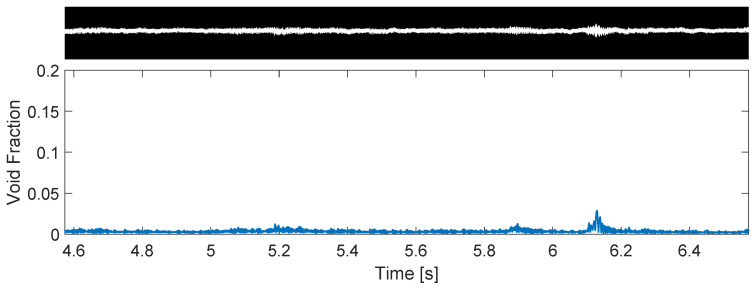
Gas–liquid distribution (**top**) and volumetric fraction of air (**bottom**) of a stable column reconstructed based on camera recordings for a water flow rate of 150 L/min and an air flow rate of 20 Ln/min. The two images correspond to the same data and share the same time axis. Gas is represented in white and liquid in black in the images.

The stable column flow pattern is marked by a centered gas core with small oscillations in the gas–liquid interface, as shown in Figure 14. Several similarities between stable columns and bubbly flows are present: both only occur for low flow rates of air, and both have a negligible intrinsic dynamics in practice.

**Figure 15:**
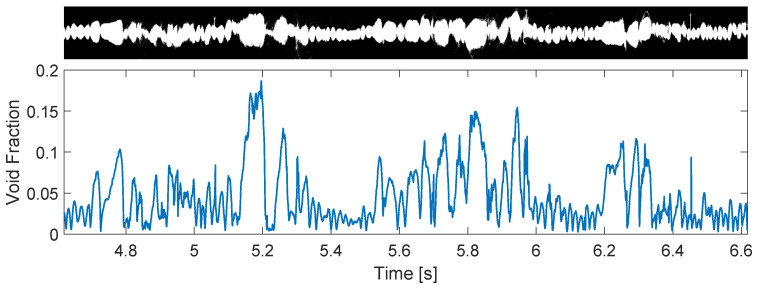
Gas–liquid distribution (**top**) and volumetric fraction of air (**bottom**) of an intermittent column reconstructed based on camera recordings for a water flow rate of 80 L/min and an air flow rate of 70 Ln/min. The two images correspond to the same data and share the same time axis. Gas is represented in white and liquid in black in the images.

The intermittent column is marked by a gas core with large oscillations in the gas–liquid interface, which often breaks into bubbles, as shown in Figure 15. This pattern is closely related to the slug flow of non-swirling flows, occurring for similar flow rates of air [46] and sharing strong intrinsic oscillations of the interface, which cannot be neglected in practical applications (e.g., when trying to capture the gas column with the pickup tube).

### 4.3. Experimental Investigation of the Swirl Effects in the Upstream Flow

**Figure 16:**
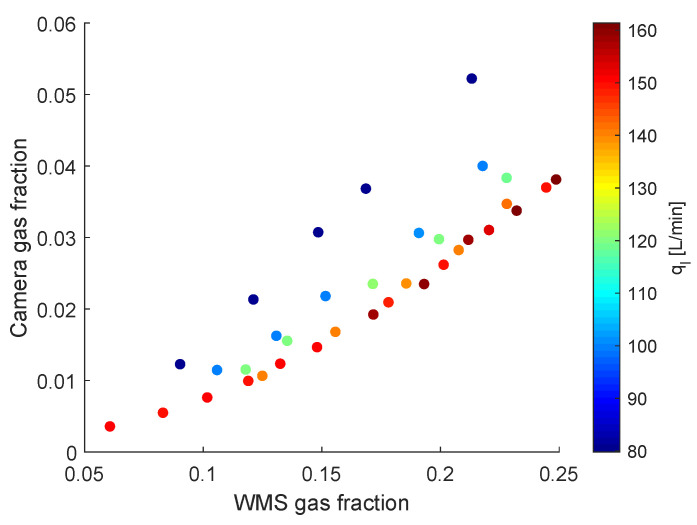
Average volume fraction of the gas core downstream of the swirl element (camera gas fraction) as a function of the average gas volume fraction upstream of the swirl element (WMS gas fraction) and the liquid flow rate ($q_{l}$).

The relation between the flows upstream and inside the inline swirl separator can be explored both in terms of external disturbances and intrinsic dynamics. To investigate the propagation of external disturbances from the upstream flow to inside the separator, a comparison between the time-averaged gas volume fraction of the upstream flow and of the gas core is presented in Figure 16.

Figure 16 shows that the swirling region has a much smaller average gas fraction than the upstream flow for the same flow rates of air and water. Moreover, the gain in the average void fraction of the non-swirling–swirling transition at the swirl element is not constant; the graph shows that a higher gain is present for low liquid flow rates (which lead to larger gas core volumetric fractions) and that the gain decreases as the liquid flow rate is increased. The effects of the liquid flow rate in the relation between void fractions seems to cease above 140 L/min, with the collapse of the experimental points in a single curve.

The dependency of the gas fraction on the liquid flow rate in the swirling section can be explained by the link between the velocity and pressure drop of each phase in the flow. The effect is illustrated considering an increase in the liquid flow rate while keeping the core size fixed. Since the area of the liquid is kept fixed, the increase in the liquid flow rate leads to an increase in the liquid velocity due to the conservation of mass (the flow rate is equal to the product between the area of the phase and its velocity), resulting in a larger pressure drop in the liquid due to an increased shear at the wall of the pipe. To satisfy the balance of forces in the flow, the pressure drop in the column of gas must follow the increase of the pressure drop of the liquid annulus. However, the pressure drop in the gas only depends on the shear at the interface between the fluids, increasing mainly with the increase in the gas velocity, and since the gas flow rate is kept fixed, this is only achievable with a reduction in the core size. Therefore, the flow has a tendency to decrease the average core size when the flow rate of water is increased, in order to balance the pressure drops of the gas and liquid phases.

**Figure 17:**
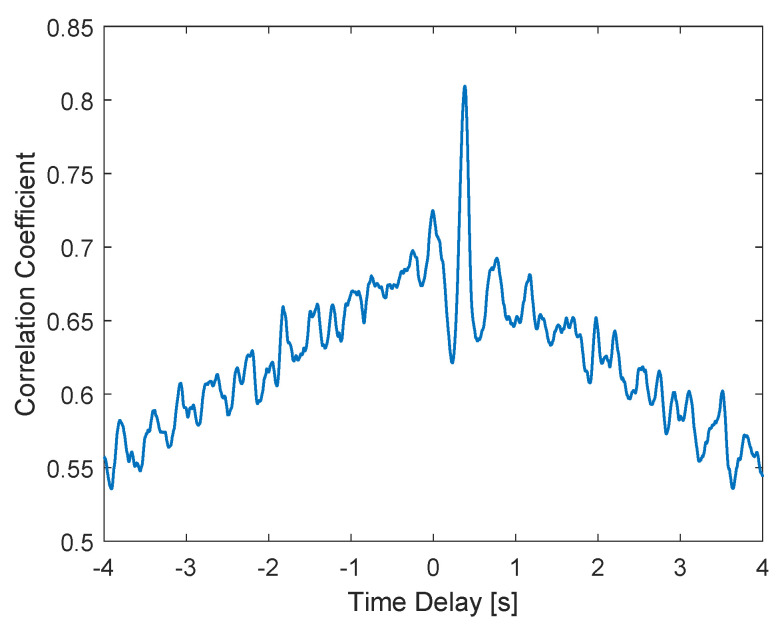
Cross-correlation coefficient between the camera and wire-mesh sensor volumetric fractions of air for a liquid flow rate of 160 L/min and an air flow rate of 170 Ln/min.

The analysis of the link between the core and the upstream flow in terms of intrinsic dynamics is performed based on the cross-correlation between the gas volumetric fraction of the gas core, obtained by processing the camera images, and of the upstream flow, obtained by the WMS measurements, for each experimental point of Appendix A. A clear peak of a relatively high cross-correlation coefficient is obtained for every point in the dataset, confirming that the core and the upstream flow are dynamically connected. Figure 17 presents one of the cross-correlation curves obtained, in which a peak of the correlation coefficient above 0.8 at 0.384 s is observed.

**Figure 18:**
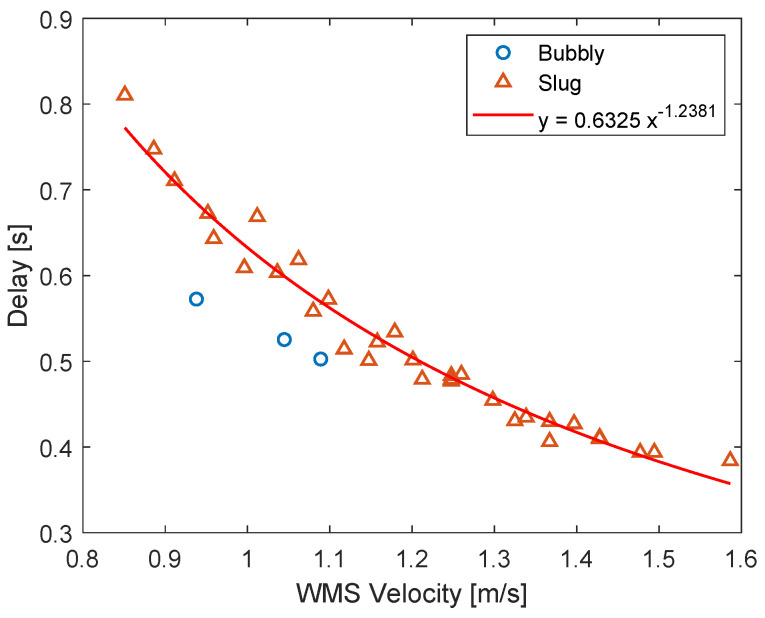
Delay that maximizes the cross-correlation between the camera gas fraction and the wire-mesh sensor gas fraction as a function of the velocity measured by the wire-mesh sensor. The symbols are used to illustrate the dependency of the relation on the flow pattern upstream of the swirl element.

The time delay that maximizes the cross-correlation between the gas fraction upstream and inside the separator depends on the flow rates of air and water used in the experiments, being linked to the average gas velocity between the WMS and the camera. Since the gas velocity in the upstream region is measured by the double-layer wire-mesh sensor, a clear trend between the delay and the WMS velocity is obtained when the quantities are plotted against each other (for all the experimental points) in Figure 18.

Figure 18 shows that the relation between the delay and the WMS velocity follows a monotonic and well-behaved curve for each flow pattern upstream of the separator. Moreover, the delay between the two signals decays with the increase in the velocity measured by the WMS sensor, which is expected: if the gas moves faster, the time required for the disturbances detected by the WMS to travel to the location recorded by the camera should be smaller.

**Figure 19:**
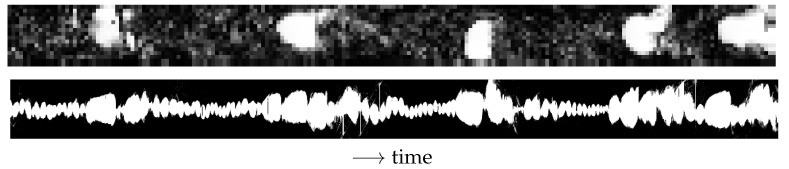
Flow reconstruction of wire-mesh sensor (**top**) and camera (**bottom**) synchronized by the delay of Figure 18 for a liquid flow rate of 80 L/min and a gas flow rate of 70 L/min. The top image shows the passage of large gas pockets (in white) with several small bubbles in between (gray pixels), and the bottom image shows sections of wide and thin core sections. The images together show the transformation of small bubbles upstream of the swirl element into regions of a thin gas core and the transformation of large gas pockets into regions of a wide gas core.

The delay of Figure 18 can be used to shift the time vector of the camera recordings with respect to the time vector of the wire-mesh sensor, synchronizing the two measurements in relation to the intrinsic dynamics. The result obtained for one experimental point is presented in Figure 19.

Figure 19 shows that the small bubbles between the large gas pockets are transformed by the swirl into a continuous thin core, while the large gas pockets are transformed into large core sections inside the separator. The multiple bubbles between the large gas pockets result in a roughly constant volume of gas reaching the swirl element, as observed by the gas volume fraction curve of Figure 13, resulting in small fluctuations in the gas–liquid interface. On the other hand, the large gas pockets cause a strong rise in the amount of air reaching the swirl element (Figure 13), and the core has to enlarge to accommodate this increase in the volume of gas. The conversion of small bubbles into a thin core and the conversion of large bubbles into a thick core is clear in the image, which also shows a complex relation between the two locations: the original gas pockets of the upstream flow becomes deformed and stretched inside the separator.

The experimental results obtained along this section confirm that the gas core inside the inline swirl separator is connected to the flow upstream of the inline swirl separator, both in relation to external disturbances (Figure 16) and intrinsic dynamics (Figure 19). Therefore, the measurements of the upstream flow in the inline swirl separator can be included in the control of the system via a feedforward term or used as measured disturbance in a model predictive controller. The results of this section obtained with the ISS are extended to quasi-1D multiphase flows in Section 6.1.

### 4.4. Numerical Simulations of the Separation

Besides the experimental approach of the previous sections, computational fluid dynamics (CFD) can be used to improve the understanding of the gas core dynamics and its connection to the upstream flow, since the technique can fully resolve the distribution of fluids from upstream of the swirl element to downstream of the pickup tube.

During the simulations, the coalescence of the bubbles present in the upstream flow into a continuous gas core inside the separator can be performed, for instance, using a hybrid CFD approach [47], which switches from the Lagrangian tracking [48] of individual bubbles to the solution of the Navier–Stokes equations inside the continuous core of gas using the volume of fluid.

**Figure 20:**
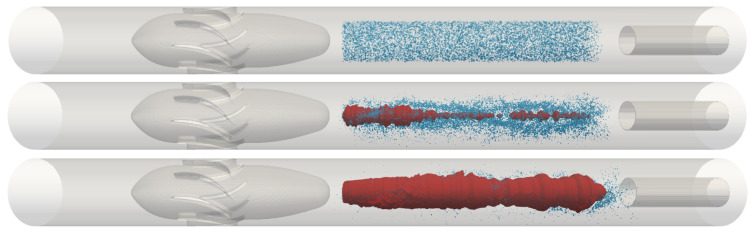
Simulation performed using the hybrid Lagrangian tracking–volume of fluid solver. The blue dots correspond to bubbles, and the red region corresponds to the coalesced core. (**Top**) t=0 s (initial condition); (**Middle**) t=0.011 s; (**Bottom**) t=0.040 s.

Figure 20 shows the results obtained in a first simulation made with the hybrid solver. A uniform mesh of spacing D/80 was adopted for the simulation after a mesh independence study [49], and the solid boundary conditions (pipe walls and swirl element) were imposed in the flow using the immersed boundary method [50]. The gas core formation was analyzed for a liquid flow rate of 217 L/min, and large eddy simulation [51] with a stochastic wall model [52] was used to model turbulence. At the current stage, bubbles are not yet injected upstream of the swirl element, but in the swirling flow.

The top image of Figure 20 shows the start of the simulation (t = 0), when 70,000 bubbles of 1 mm are inserted between the swirl element and the pickup tube with the same velocity as the liquid phase. Due to the swirl, the bubbles are pushed towards the center of the pipe while moving in the axial direction, and a thin gas core is quickly formed inside the separator (middle image). The coalescence process continues, and the gas core grows in size (bottom image). Further in the simulation, the gas core presents instabilities in the interface similar to the ones observed in experiments with intermittent columns [49].

Figure 20 shows a qualitative agreement between the gas core observed in the experiments and in the CFD simulations, illustrating the potential of the technique for the analysis of the gas core dynamics. The following steps for a comparison between the experiments and the simulation is the implementation of a continuous injection of gas upstream of the swirl element, in order to analyze the propagation of disturbances from the upstream flow to inside the separator both qualitative and quantitatively.

## 5. The Real-Time Control of Multiphase Flows

A proportional–integral (PI) controller was implemented in the inline swirl separator to evaluate the possibilities and limitations of tomography-based real-time control of multiphase flows, providing a background for the discussions of Section 6.

**Figure 21:**
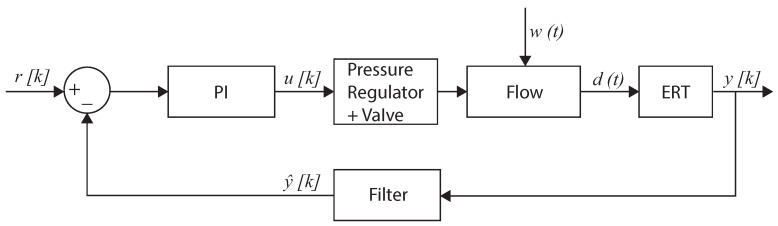
Closed loop block diagram with the ERT sensor and the PI controller. d(t) is the gas core size inside the installation (continuous-time signal), which is sampled by the ERT system as y[k] (discrete-time signal). The ERT signal is filtered into $\hat{y}$, which is compared to a core-size reference (*r*), and the difference between the two quantities is used by the PI controller to compute the controller output, *u*. The variable *w* summarizes the unsteady effects of the core, due to the propagation of intrinsic dynamics from the upstream flow and to external process disturbances.

The PI controller uses the ERT measurements of gas core size to compute control actions in the pickup tube valve, as shown in Figure 6; the wire-mesh sensor and camera were used to illustrate the propagation of disturbances and intrinsic dynamics in the separator and were not used in the control experiments. The controller was implemented according to the block diagram of Figure 21.

The gas core inside the inline swirl separator is affected by changes in the pickup tube valve and by intrinsic dynamics and process disturbances that propagate from the upstream flow to the core (Section 4). In the block diagram of Figure 21, the pickup tube valve position is connected to the controller output of pressure in the cavity of the valve, u[k], and the unsteady effects of the core (both due to intrinsic dynamics and process disturbances) are summarized into the variable w(t). The gas core size, d(t), which is connected to both u(t) and w(t), is sampled by the ERT system at 10 Hz as y[k], where *k* are the samples of the sensor. Due to the intrinsic dynamics of the core, the ERT signal must be filtered into $\hat{y}\left[k\right]$ to ensure the stability of the PI controller implemented in the loop, which is designed to react to slow changes in the flow caused by external process disturbances. The filtered core size is compared to a reference value, r[k], by the PI controller, which uses the difference between the signals to compute the controller output, u[k], which is sent to the pressure regulator connected to the pickup tube valve by LabVIEW during the same sample *k* almost instantaneously. Between the ERT samples, a zero-order hold of u[k] is performed by LabVIEW, i.e., u(t)=u[k] for $kT_{s}\leq t<\left(k+1\right)T_{s}$.

**Figure 22:**
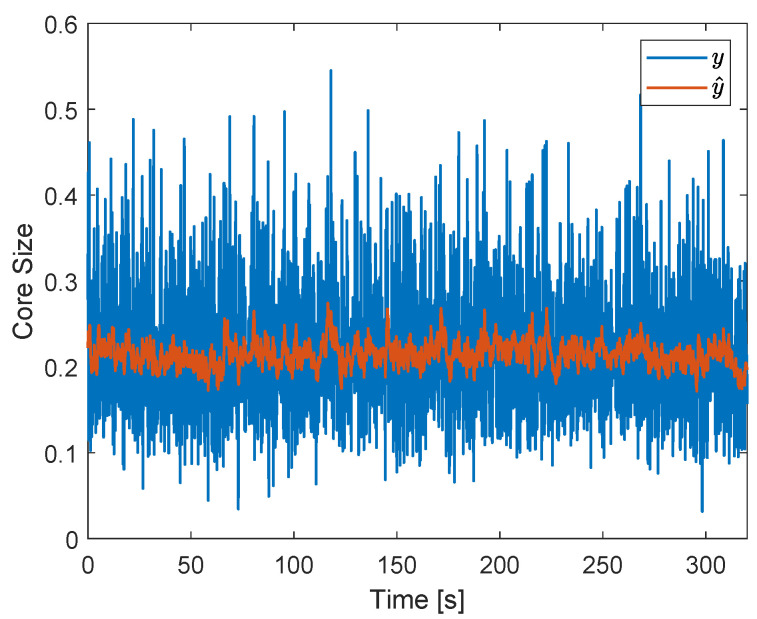
Core size obtained for an air flow rate of 110 Ln/min and a water flow rate of 113 L/min at fixed valve positions, which maximize $\eta_{m}$. In blue: original ERT signal. In orange: output of the low-pass filter ($\omega_{f}=1$ rad/s). Signals with standard deviation σ(y)=0.0724 and $\sigma (\hat{y})=0.0146$.

The reference of the controller, *r*, is chosen based on the average core size measured by the ERT when the pickup tube and water outlet valves are at fixed positions, which maximizes the mean efficiency of separation ($\eta_{m}$), for a certain pair of flow rates. Due to limitations in its spacial resolution, the ERT system requires relatively large gas cores inside the separator, which only occur for relatively large flow rates of air. Then, after some tests, the water and air flow rates were chosen, respectively, as 113 L/min and 110 Ln/min, and $\eta_{m}$ was maximized when both valves were at intermediary positions. The combination resulted in the ERT measurement of the gas core size of Figure 22, which shows an unsteady gas core of average size 0.21. The condition also resulted in slug flow upstream of the separator and efficiencies: $\eta_{a}=88\%$, $\eta_{w}=74\%$ and $\eta_{m}=81\%$.

Due to the (intrinsic) gas core size oscillations observed in Figure 22, which have a large amplitude and high frequency (in the order of Hz), a discrete low-pass filter was used to filter the ERT signal. A cutoff frequency $\omega_{f}=1$ rad/s was chosen for the filter, which was implemented in discrete-time based on the backward Euler approximation:

(11) $$\hat{y}\left[k\right]=\frac{\omega_{f}T_{s}y\left[k\right]+\hat{y}\left[k-1\right]}{1+\omega_{f}T_{s}},$$

where *k* is the sample index, y[k] is the output of the ERT sensor, and $\hat{y}\left[k\right]$ is the filtered core size, used to compute the control actions. The sample time of the controller and ERT was $T_{s}=0.1$ s. The orange curve of Figure 22 shows the result obtained when applying the filter to the gas core size signal observed in the system without external disturbances or control actions (blue curve).

The PI controller was implemented in discrete-time with the integral term approximated by the trapezoidal rule:

(12) $$u\left[k\right]=K\left(e\left[k\right]+\frac{T_{s}}{T_{i}}\frac{e[k]+e[k-1]}{2}\right),$$

where *K* is the gain of the controller, $T_{i}$ is the integral time and, $e=r-\hat{y}$. After a trial and error tuning of the controller, targeting a fast response of the valve while maintaining a stable gas core size, the parameters of the controller were set to K=1.5 and $T_{i}=2\;s$. The controller setpoint was chosen as r=0.2, slightly below the mean core size of Figure 22.

### 5.1. Control in the Absence of External Process Disturbances

**Figure 23:**
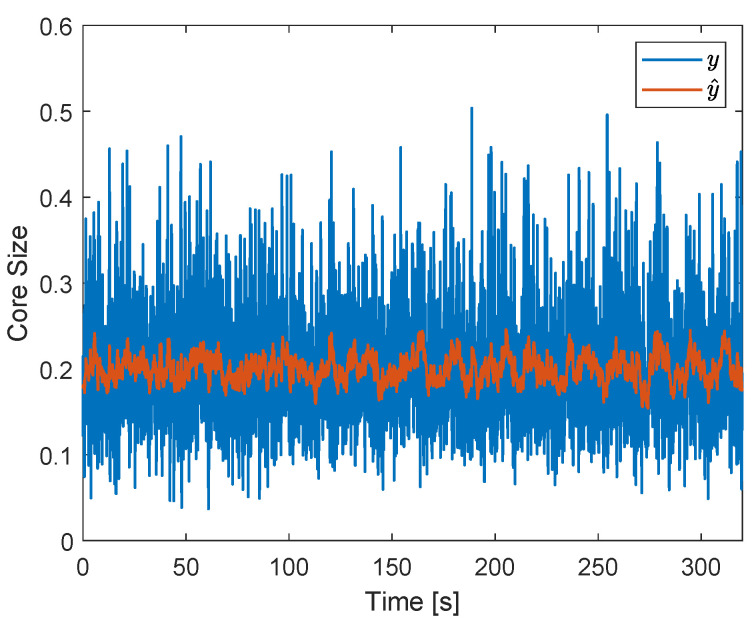
Controlled core size in the absence of external disturbances. In blue: original ERT signal. In orange: output of the low-pass filter ($\omega_{f}=1$ rad/s). Signals with standard deviation σ(y)=0.0719 and $\sigma (\hat{y})=0.0154$.

Since the control actions are based on the filtered ERT signal, which removes the majority of the intrinsic dynamics of *y*, it is expected that it has a minor impact on the system in the absence of external process disturbances. Figure 23 shows the controlled gas core size obtained when no external disturbances are applied to the system.

The controller setpoint of 0.2 for the gas core size shifts the original mean core size of Figure 22 from 0.21 to 0.2 in Figure 23, while maintaining roughly the same amplitude of the intrinsic dynamics in the two conditions: the standard deviations of the core size (blue curves) are σ(y)=0.0724 (original) and σ(y)=0.0719 (controlled). A small increase in the standard deviation of the filtered core size (orange curves) is observed for the controlled case ($\sigma (\hat{y})=0.0154$) in relation to the uncontrolled case ($\sigma (\hat{y})=0.0146$), indicating an increase in the low-frequency oscillations of the core due to the controller.

The control of the system in the absence of external disturbances leads to the efficiencies $\eta_{a}=92\%$, $\eta_{w}=70\%$, and $\eta_{m}=81\%$. When compared to the uncontrolled case ($\eta_{a}=88\%$, $\eta_{w}=74\%$ and $\eta_{m}=81\%$), an increase of 4% in the air efficiency, a decrease of of 4% in the water efficiency, and as a consequence, the same mean efficiency are observed. The efficiency behavior observed is a consequence of the reduction of the mean core size, which leads to the capturing of more air (increasing its efficiency) and more water (lowering its efficiency) by the pickup tube.

The results obtained show that, if the intrinsic dynamics are filtered out in the control loop and no external process disturbances are present in the system, the controller has a minor impact on the distribution of phases (Figure 23) and on the mean efficiency of separation. However, since the controller is able to bring the mean core size towards the reference, it can be used to improve the efficiency of one of the fluids (in this case, air) or to bring the system from different initial conditions to its operating point.

### 5.2. Control in the Presence of Process Disturbances

**Figure 24:**
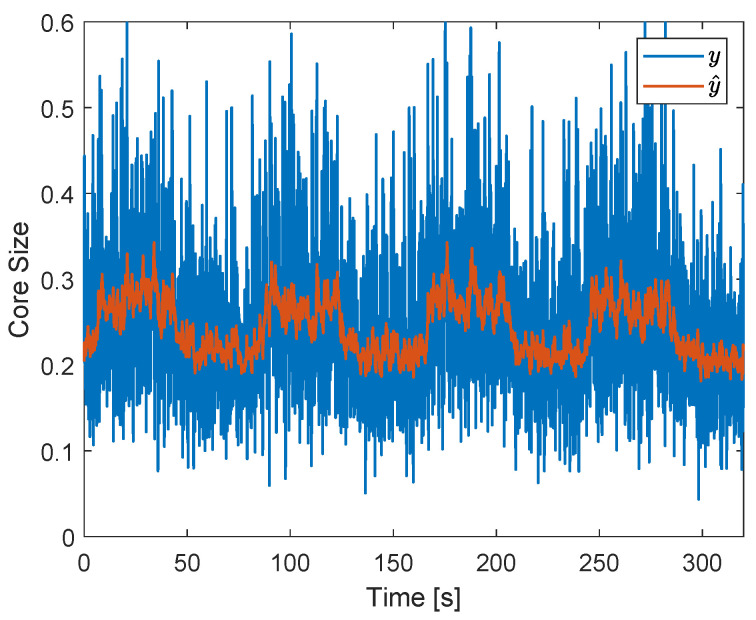
Uncontrolled core size obtained for a square wave disturbance in the air flow rate of the experimental facility. In blue: original ERT signal. In orange: output of the low-pass filter ($\omega_{f}=1$ rad/s). Signals with standard deviation σ(y)=0.0902 and $\sigma (\hat{y})=0.0323$.

A square wave in the gas flow rate was applied in the system to evaluate the feedback controller in relation to the rejection of (external) process disturbances. During the experiments, the air flow rate ($q_{g}$) was alternated between 110 and 140 Ln/min every 40 s, which resulted in the gas core size of Figure 24 when the ERT-based controller was not implemented in the loop.

Figure 24 shows that, without the controller, the filtered core size follows the changes in the air flow rate of the installation, alternating between around 0.21 (when $q_{g}=110$ Ln/min) and around 0.28 (when $q_{g}=140$ Ln/min) during every cycle of the disturbance. A higher standard deviation is observed in the raw (σ(y)=0.0902) and filtered ($\sigma (\hat{y})=0.0323$) gas core size signals in relation to the operating point of Figure 22 (σ(y)=0.0724 and $\sigma (\hat{y})=0.0146$), associated with the excitation of the extrinsic flow dynamics by the disturbance.

The disturbance results in $\eta_{a}=76\%$, $\eta_{w}=75\%$, and $\eta_{m}=75\%$. The relatively low air efficiency, when compared to the (undisturbed) operating point of Figure 22 ($\eta_{a}=88\%$, $\eta_{w}=74\%$, and $\eta_{m}=81\%$), is a consequence of the relatively large gas core formed in the separator when the air flow rate is at 140 Ln/min, which results in the capture of more air by the liquid outlet during the intrinsic fluctuations of the core. Surprisingly, the water efficiency is roughly the same in both the undisturbed and disturbed scenario; it was expected that less water would be captured by the pickup tube when a larger core is present in the system. The behavior shows the non-trivial connection between the filtered core size and the efficiency of the process due to the intrinsic dynamics, explored in Section 6.3. Overall, the disturbance causes a decay of 6% in the mean efficiency of separation in relation to the condition without disturbances or valve actions.

**Figure 25:**
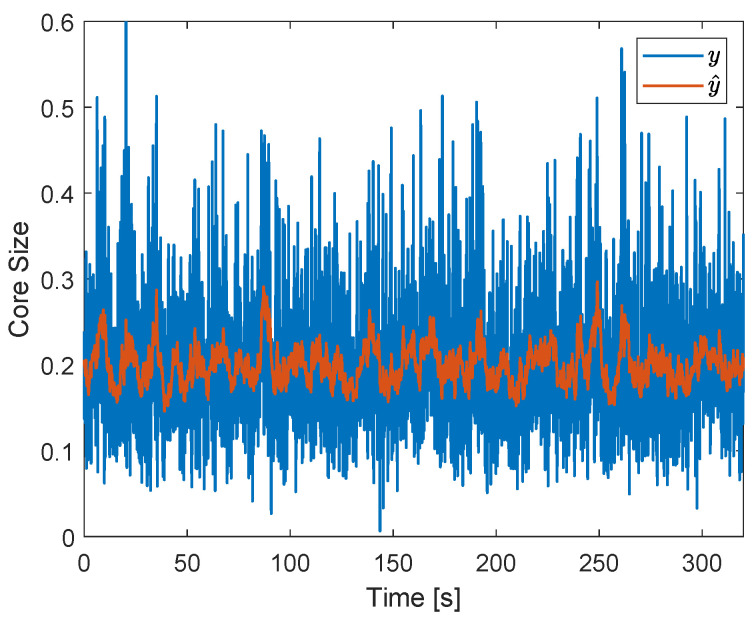
Controlled core size in the presence of a square wave disturbance in the air flow rate of the experimental facility. In blue: original ERT signal. In orange: output of the low-pass filter ($\omega_{f}=1$ rad/s). Signals with standard deviation σ(y)=0.0801 and $\sigma (\hat{y})=0.0227$.

Figure 25 shows the core size obtained in the presence of the square wave disturbance when controlling the separation. The figure shows that the filtered core size is kept around 0.2 (controller setpoint) during the entire measurement, independent of the changes in the air flow rate of the installation. An increase in the fluctuations of core size can be observed for the controlled case (σ(y)=0.0801) in relation to the operating point without disturbances (Figure 22, σ(y)=0.0724), due to a larger amplitude of the gas core intrinsic dynamics when $q_{g}=140$ Ln/min.

The control of the core for the disturbance in the gas flow rate resulted in $\eta_{a}=93\%$, $\eta_{w}=63\%$, and $\eta_{m}=78\%$. In comparison to the uncontrolled case with the (same) disturbance ($\eta_{a}=76\%$, $\eta_{w}=75\%$, and $\eta_{m}=75\%$), a gain of 17% was obtained in the air efficiency and a decay of 12% was obtained in the water efficiency, resulting in a gain of 3% in the mean efficiency. Despite a relatively small gain in $\eta_{m}$, a notorious increase in $\eta_{a}$ was achieved by the controller, which can be explored in applications where capturing the air in the pickup tube is more important than having an optimal mean separation of the phases. The main results obtained along this section are summarized in Table 1.

A successful proof of concept of tomography-based feedback control of the distribution of phases in multiphase flows was obtained by the ERT-based PI controller, which was able to keep the (filtered) gas core size around its setpoint in the presence of external process disturbances in Figure 25. However, the results obtained suggest that controlling the (filtered) distribution of phases is different from controlling the efficiency of the process; the process improvement introduced by the ERT-based controller is strongly dependent on the efficiency criteria selected: a substantial gain is achieved if the process focuses on the capture of air by the pickup tube, but a small gain is achieved if the process focuses on the average separation of the fluids.

## 6. Perspective

This section extends the results of Section 4 and Section 5 to the context of quasi-1D multiphase flows, in order to illustrate better the possibilities and limitations of tomography-based real-time control of multiphase processes.

### 6.1. Upstream Flow and Predictive Controllers

Section 4.3 explored the connection between the flow upstream of the inline swirl separator and the gas core formed inside the equipment, in relation to extrinsic disturbances and intrinsic dynamics: (i) in terms of disturbances, it was shown that the void fraction of the gas core is connected to the average gas fraction of the upstream flow; (ii) in terms of intrinsic dynamics, it was shown that the passage of large bubbles in the upstream flow causes oscillations in the gas core inside the separator, with the delay between the two effects connected to the gas velocity in the upstream region (measured by the wire-mesh). Both effects are not particular to the inline swirl separator, but present in every quasi-1D multiphase flow system.

In terms of the average (i.e., for the external disturbances), changes in the flow rates of the system lead to new interactions between the phases, both in the upstream region and inside the equipment. As a consequence, new volume fractions are obtained at each location to satisfy the local changes in the balance of forces. Since the momentum equation of the flow is strongly nonlinear (Navier–Stokes), both in the upstream region and inside the equipment, the relation between the two locations is non-trivial; for instance, it depends on the liquid flow rate for the inline swirl separator.

A direct connection between the upstream flow and the flow inside the equipment takes place in relation to fluctuations (i.e., for the intrinsic dynamics); if the fluctuations are small, the system approaches a linear behavior and the link between the two regions can be analyzed based only on conservation of mass. Industrial systems are typically dominated by convection, such that the differential conservation of mass of each phase in the mixture is given by

(13) $$\frac{\partial \rho \varepsilon }{\partial t}+\nabla \cdot \left(\rho \varepsilon u\right)=0,$$

where ρ is the density of the phase, ε its volumetric fraction, and u its velocity field.

In most applications, the flow velocity is considerably smaller than the speed of sound, and the gradients of pressure and temperature are small in comparison to their absolute values, such that the phases can be considered incompressible (i.e., density is not a function of time or position).

The velocity field of Equation (13) is given by the (nonlinear) Navier–Stokes equations. However, in quasi-1D flows (e.g., pipe flows), the equation can be simplified based on the hypothesis of plug flow:

(14) $$\frac{\partial \varepsilon }{\partial t}+\frac{\partial \varepsilon u}{\partial s}=0,$$

where *s* is the position along the flow direction.

If the spatial derivative of the velocity along the flow direction is small, which is a good hypothesis if there is no gradual change in the system geometry, the equation is further simplified to

(15) $$\frac{\partial \varepsilon }{\partial t}+u\frac{\partial \varepsilon }{\partial s}=0$$

The solution of Equation (15) is given by ε(t+s/u)=constant, which is equivalent to a delay between the location of interest and the inlet, $\varepsilon^{\prime }\left(t\right)=\varepsilon_{inlet}^{\prime }\left(t-\tau \right)$, with τ=Δs/u.

**Figure 26:**
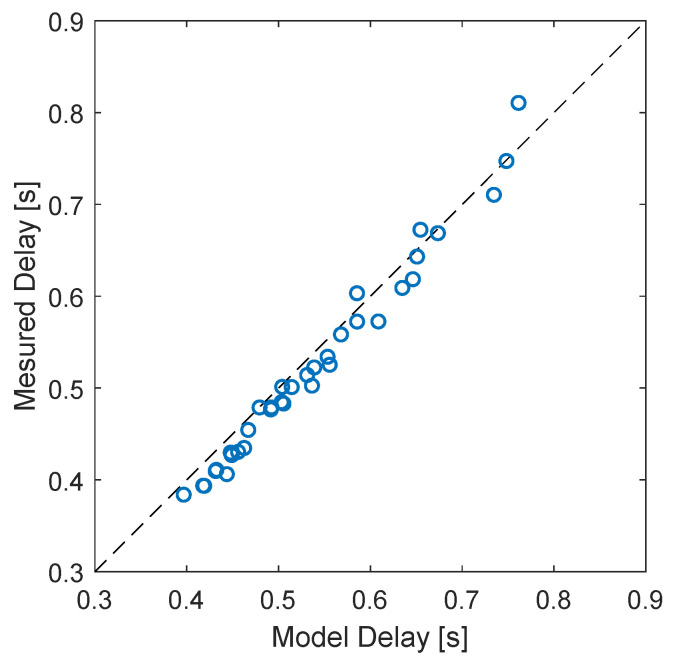
Comparison between the delay obtained cross-correlating the signal of the camera and WMS (measured delay), previously shown in Figure 18, and the delay estimated based on the conservation of mass (model described in Appendix B). The perfect match between the model and experiments is represented by the dashed line.

To illustrate how powerful the solution of Equation (15) is, the delay measured between the gas volumetric fraction signals of the wire-mesh sensor and camera in the ISS application for all the experimental points of Appendix A, previously presented in Figure 18, is compared to the exact solution obtained from the conservation of mass (detailed in Appendix B) in Figure 26.

Figure 26 shows a great match between the model and the experiments, confirming that the fluctuations of the volume fraction inside industrial equipment are always connected to the upstream flow via the conservation of mass. As a consequence, controllers taking into account the measurement of the upstream flow can be designed to react faster than pure feedback approaches, which is particularly interesting when targeting the control of intrinsic dynamics. For instance, a feedforward action could be added to the feedback controller of Figure 21 to act on the pickup tube valve based on the measurement of the wire-mesh sensor installed upstream of the swirl element. Another option would be to include the wire-mesh measurement in a model predictive controller, which would use it to predict the gas core at the pickup tube and use the prediction to compute the control actions in the valve. The model linking the upstream conditions to the gas core can be either analytic, based on the conservation of mass (e.g., in Appendix B), based on the conservation of mass and momentum (e.g., in [53]), or based on empirical fits (e.g., the power law of Figure 18).

### 6.2. The Time Scales of Multiphase Flow Processes and the Design of Real-Time Controllers

In Section 5, a feedback controller was implemented in the ISS to reject external process disturbances in the distribution of fluids (gas core size) near the pickup tube, achieving successful results. During the implementation, a low-pass filter was used to eliminate the majority of the intrinsic dynamics present in the ERT measurements of the gas core size from the calculations of the controller. Naturally, by removing most of the intrinsic dynamics of the process, the controller was only able to react to extrinsic disturbances.

The choice to exclude the intrinsic dynamics from the control actions, by filtering the ERT signal, was mainly due to the challenge of designing a controller able to react fast enough to the intrinsic dynamics, which dominate the gas core size in the frequencies of around 1Hz. The same challenge is found in many industrial processes due to:

- Safety: Fast actions in the flow, matching the time scales of the intrinsic dynamics, can result in dangerous situations, especially when dealing with liquids. As liquids are incompressible and have large densities, sudden changes in valves can cause water hammer effects and pressure spikes in the system, which can damage the equipment and result in cracks and leakages.
- The high inertia of industrial equipment: Industrial equipment typically stores large masses of liquid, which must be accelerated whenever a change is made in the boundary conditions of the system (e.g., a change in the opening of a valve). Therefore, even if an actuator fast enough to match the time scales of the intrinsic dynamics can be used in the application, the high inertia of the system often leads to a flow response too slow in relation to the intrinsic dynamics.
- Nonlinearities and robustness: Multiphase flows are nonlinear by nature, and nonlinearities in industrial equipment are also common. For instance, the control valves of the ISS used in this study have a strong hysteresis and a nonlinear impact on the flow. Therefore, it is hard to design a controller that is stable in practice and operates in the time scales of the intrinsic dynamics, especially without a careful analysis of the physics behind the process.

Although a low-pass filter can be used to remove the intrinsic dynamics from the calculations of the controller, it still plays a large (if not major) role in the performance of the equipment, as detailed in the next subsection. Therefore, whenever possible, it is still interesting to act on them. To analyze the possibility of controlling the intrinsic dynamics, a comparison between its time scales and the time scales of the system response (in closed loop) must be made, taking into account the limitations of the actuator (both physical and in terms of safety) and the delays of the process. Moreover, the addition of a feedforward controller and/or the consideration of the nonlinearities of the process can improve considerably the chances of success of controlling the intrinsic dynamics.

### 6.3. The Effects of the Intrinsic Dynamics in the Operating Point of the System and Controller Performance

A setpoint of 0.2 was adopted for the normalized core size in Section 5, based on the average core size obtained at operating conditions that maximize the mean efficiency. One can note that 0.2 is considerably smaller than the normalized inner diameter of the pickup tube, 0.44, a natural choice of the setpoint for a perfect separation: if the gas core is centered and has the same size as the pickup tube, then all the air is captured by the outlet and all the water flows to the liquid outlet. Although the gas core is measured 0.9D upstream of the tip of the pickup tube and continues to enlarge before being captured, its size increase between the ERT and the pickup tube is certainly below 0.24D.

The large gap between the core size observed at maximum mean efficiency and the pickup tube size occurs due to the intrinsic dynamics of the gas core. Since the gas core size presents large-amplitude fluctuations (Figure 22), air is always captured by the liquid outlet unless a considerably thin core is formed inside the separator. However, this condition results in the capture of much water by the pickup tube and a low water efficiency. From the liquid side, a wide (average) gas core must be present in the separator to ensure that most of the water reaches the liquid outlet despite the fluctuations, but this also leads to more air reaching the liquid outlet during the fluctuations and a low air efficiency. Therefore, it is impossible to simultaneously improve both the air and water efficiencies without the control of the intrinsic dynamics, and the mean efficiency can never be substantially improved by controlling the process.

Another important aspect of the intrinsic dynamics is that they are not completely independent of the extrinsic process disturbances. The intrinsic dynamics are a consequence of the nonlinear physics of the flow for a set of boundary conditions, and whenever the boundary conditions are changed, new balances of mass and momentum are achieved in the system, resulting in different intrinsic dynamics. The connection between intrinsic dynamics and external disturbances can be illustrated, for instance, considering an increase in the gas flow rate of a system that is designed to operate in the bubbly regime, causing it to transition to slug. In this example, the intrinsic dynamics of the system are initially negligible due to bubbly flow, and the external disturbance leads to strong intrinsic dynamics in the process due to the transition to slug.

This nonlinear relation between intrinsic dynamics and process disturbances causes complications when targeting improvements in the performance of multiphase flow processes without the control of the intrinsic dynamics. Since the efficiency of the process is strongly connected to the intrinsic dynamics and the intrinsic dynamics are related to external disturbances, efficiency is not only a function of the filtered distribution of phases, but also of the disturbance itself. Therefore, even if the former is controlled using tomography, there is still an impact of the latter in the performance of the process due to its effects in the intrinsic dynamics.

### 6.4. Application-Specific Tomography and the Monitoring of Intrinsic Dynamics

An additional limitation to the control of intrinsic dynamics, not previously mentioned in Section 6.2 and particular to the context of tomography-based control, is the temporal and spatial resolution of tomographic techniques that operate in real-time.

In general, due to the step of image reconstruction, tomographic techniques that are precise in space are computationally demanding and, therefore, slow, and tomographic techniques that are fast are not well resolved in space. Naturally, a good spatial resolution is required to control the process based on the measurement of the distribution of phases, and the temporal resolution of the sensor should be sufficient to capture all the relevant time scales of the process.

External disturbances are relatively slow, and therefore, it is possible to reject them using classical tomographic reconstruction techniques, which are time consuming, together with slow actuators and slow process controllers. However, intrinsic dynamics occur at frequencies typically above the temporal resolution of the tomographic reconstruction of the flow, and alternatives to speed up the measurement must be developed.

The main limitation in the temporal resolution of tomography (in particular, soft-field tomography) comes from the software, which solves the inverse problem, and not from the hardware, which performs the acquisition of data (e.g., the voltage–current measurements of ERT). Therefore, if the inverse problem is replaced by fast reconstruction schemes, the limitation in the software of tomography is removed and the system can be used to measure the distribution of phases close to the maximum hardware frequency.

The computationally demanding inverse problem solved by the software is based on physical laws (e.g., Maxwell equations), having a generalist characteristic: the set of equations solved by the inverse problem is valid for any application of the sensor. However, many multiphase flow processes have well-defined flow distributions, including the inline swirl separator: the gas core is a single object inside the ERT domain, with a roughly circular cross-section. Therefore, instead of reconstructing the whole distribution of conductivity in the ERT cross-section, based on the general time-consuming inverse problem, and then extracting the gas core from the image, a search for the size and position of the gas core directly based on the (raw) measurements of current can be performed [36]. In comparison to the original problem of hundreds or thousands of variables (electrical conductivity of a grid of points), the new problem, valid only for the inline swirl separator, has three variables and can be solved almost instantaneously.

Naturally, the distribution of phases in multiphase flows can be considerably more complex than the two well-defined regions of the gas core and liquid annulus of the ISS. For instance, bubbly flows have multiple elements (bubbles) distributed across the cross-section of the pipe, and reconstructing each single bubble based on raw data with simple relations is most likely impossible. However, the interest in practical applications is rarely on the full reconstruction of the flow, but in obtaining a small set of variables that summarize its most relevant aspects; for instance, the gas volume fraction and the bubble size distribution are the most important variables of bubbly flows.

A small set of process variables measured by tomography is particularly interesting for control applications, since it makes the choice of the actuator(s) and the design of the controller more intuitive. In the ISS, it is easy to understand the effect of the pickup tube valve in the size of the gas core and to chose a controller setpoint. However, it would be complicated to understand the effects of the valve in the whole distribution of phases in the ERT section and to define a reference for each point/pixel in the images reconstructed provided by it. Moreover, since there is a single actuator in the system (pickup tube valve), it is probably impossible to control all the states of the process (gas volume fraction of each pixel in the ERT image).

The number of relevant multiphase flow variables is always smaller than the number of raw data measurements obtained by the tomographic system, and application-specific algorithms can be designed to filter the relevant measurements from the raw data and link them to process variables, either physically based, as in [36], or using strategies designed to handle complex relations between variables (e.g., machine learning).

Besides removing the software limitations of tomography, application-specific algorithms can be used to limit the data acquisition of tomographic equipment to the relevant measurements (in the ISS example, 12 current measurements rather than 240), which also allows the hardware to operate faster. As a consequence, the measurement speed of the technique can be improved by orders of magnitude, and tomography can be applied in real-time measurements of the intrinsic dynamics of multiphase flows.

## 7. Conclusions

The main objective of this paper was to evaluate the potential application of tomography in the real-time control of multiphase flows, which was performed in two steps: First, an electrical resistance tomography-based PI controller was used to reject external disturbances in the distribution of phases of an inline swirl separator (Section 5). Then, the results obtained with the separator were extended to the context of quasi-1D multiphase flows in Section 6. The paper showed that:

- The distribution of phases in multiphase flows has two unsteady components: (i) the intrinsic dynamics, connected to the multiphase flow patterns, and (ii) the extrinsic dynamics, associated with external process disturbances.
- The intrinsic dynamics of the distribution of phases inside industrial equipment is connected to the intrinsic dynamics of the flow upstream of the equipment, due to the conservation of mass. Therefore, feedforward actions or model predictive controllers can be designed based on the measurement of the inlet of the equipment, either using tomographic or non-tomographic (as wire-mesh sensor) techniques.
- The choice between controlling the intrinsic dynamics of the flow, or limiting the control to external process disturbances, must be performed based on the knowledge of the time scales of the intrinsic dynamics, the temporal resolution of the sensor, and the time scales of the system in relation to control actions.
- If not controlled, the intrinsic dynamics strongly influences the choice of the operating point of the system and the controller setpoint, weakens the link between the filtered distribution of phases and performance (e.g., efficiency), and limits the improvements in performance that can be achieved by controlling the process.
- Classical tomographic reconstruction techniques are too slow to monitor the intrinsic dynamics of multiphase flows in real-time, and application-specific algorithms must be developed to improve the temporal resolution of the technique in control applications.
- Tomography can be applied in the real-time control of the distribution of phases of quasi-1D multiphase flows, illustrated in this paper by the successful rejection of external disturbances in the gas core by the ERT-based PI controller implemented in the inline swirl separator.
- The control multiphase flow systems using tomography has the potential of substantially increasing the performance of the process; when rejecting external process disturbances, the ERT-based PI controller implemented in the inline swirl separator increased the capture of air by the pickup tube from 76% to 93% of the total flow rate injected in the system (an increase of 17%) and the mean efficiency of the process from 75% to 78%.

## Figures and Tables

**Table 1:** Standard deviation of core size and efficiencies measured in the experiments of Section 5.

Experiment	σ(y)	$\sigma (\hat{y})$	$\eta_{a}$	$\eta_{w}$	$\eta_{m}$
Operating Point (Figure 22)	0.0724	0.0146	88%	74%	81%
Control of Intrinsic Dynamics (Figure 23)	0.0719	0.0154	92%	70%	81%
Uncontrolled Disturbance (Figure 24)	0.0902	0.0323	76%	75%	75%
Control of Disturbance (Figure 25)	0.0801	0.0227	93%	63%	78%

**Figure A1:**
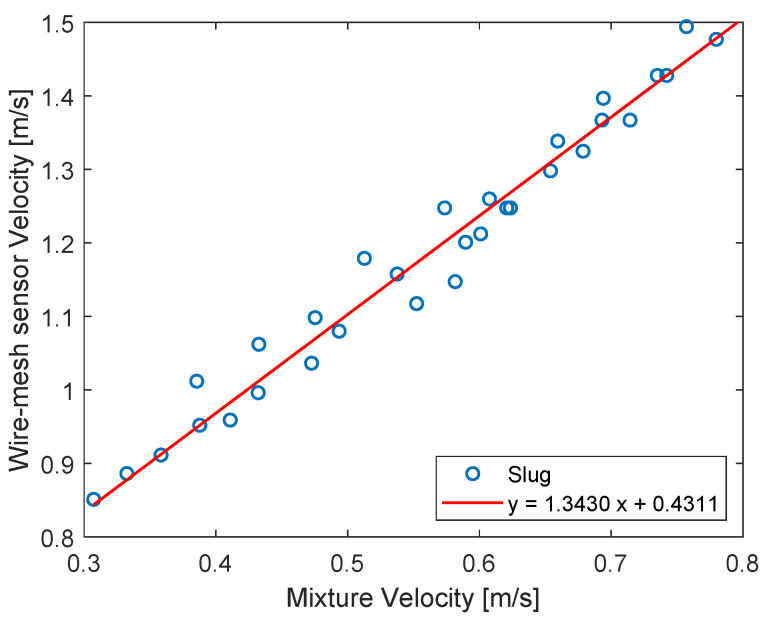
Comparison between the gas velocity and the mixture velocity in the WMS location for slug flows. The flow rate of air used in the calculation of the mixture velocity is obtained from the mass flow rate injected in the system and pressure measurements near the WMS.

**Figure A2:**
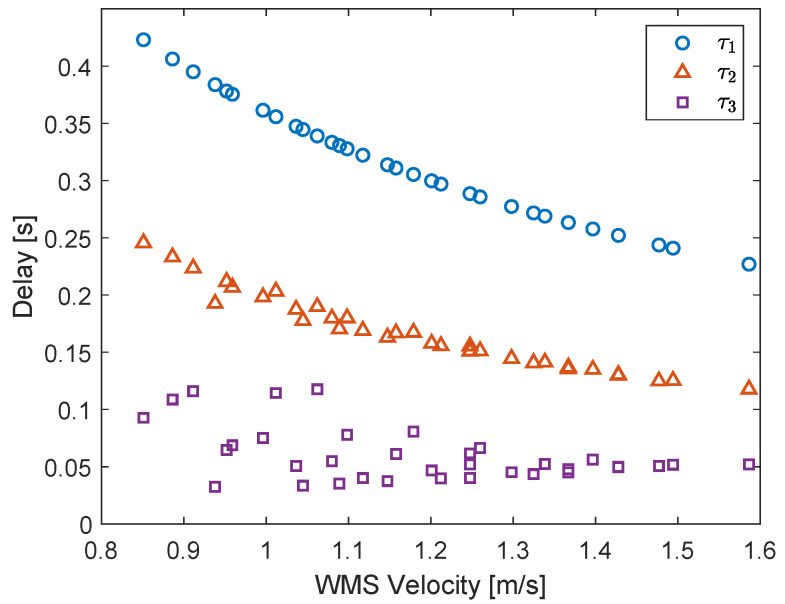
Components of the delay in the void fraction signal detected by the wire-mesh sensor and by the camera, predicted according to the model described in Appendix B for all the experimental points of Appendix A. The results are presented as a function of the gas velocity measured by the double-layer WMS, since it summarizes the gas and liquid flow rates into a single variable. $\tau_{1}$ corresponds to the time required for the gas to move between the WMS and the swirl element; $\tau_{2}$ corresponds to the time required for the gas to cross the swirl element; $\tau_{3}$ corresponds to the time required for the gas to travel between the outlet of the swirl element and the camera.

## Data Availability

Not applicable.

## Abbreviations

The following abbreviations are used in this manuscript:

mv	Measured value
CFD	Computational fluid dynamics
ERT	Electrical resistance tomography
ISS	Inline swirl separator
PI	Proportional–integral
UDP	User datagram protocol
WMS	Wire-mesh sensor

## Appendix A. Experimental Points of the Upstream–Core Connection

Thirty-five combinations of liquid and gas flow rate were used in the experiments of Section 4, summarized in Table A1. All the experiments were performed with the liquid outlet valve partially closed and the pickup tube valve fully open.

**Table A1:** Gas and liquid flow rates used in the study of the connection between the gas core and the upstream flow. In the table, Ln/min stands for normal liters per minute, defined as the volumetric flow rate of gas calculated from measurements of mass flow rate considering the density of air at 0 °C and 1 atm (Section 3.1).

Flow Rate of Water (L/min)	Flow Rate of Air (Ln/min)
80	20, 30, 40, 50, 70
100	30, 40, 50, 70, 90
120	40, 50, 70, 90, 110
140	50, 70, 90, 110, 130
150	20, 30, 40, 50, 60
150	70, 90, 110, 130, 150
160	90, 110, 130, 150, 170

## Appendix B. Estimation of the Wire-Mesh Sensor–Camera Delay

The delay between the gas volume fraction detected by the wire-mesh sensor installed upstream of the swirl element and the gas volume fraction inside the inline swirl separator in the area recorded by the camera can be approximated as the sum of three individual delays, which are calculated based on the local gas velocity and geometry:

(A1) $$\tau =\sum_{i=1}^{3}\frac{\Delta s_{i}}{u_{i}}$$

Following the dimensions of the loop presented in Figure 7, each component of the delay is calculated considering that:

i. The gas moves at the speed detected by the wire-mesh sensor ($V_{wms}$) between the wire-mesh sensor and the swirl element, leading to $\tau_{1}=4.4\;D/V_{wms}$.
ii. The body of the swirl element (se) causes a contraction of the flow, which leads to a mixture velocity inside the vanes of the swirl element larger than in the pipe below it. The mixture velocity is defined as the total flow rate divided by the cross-sectional area of the flow. Since the total flow rate is the same both upstream and inside the vanes, the mixture velocity of the two locations are connected by:
  (A2) $$V_{m,se}=\frac{A}{A_{se}}V_{m},$$
  where *A* is the cross-sectional area of the pipe, $A_{se}$ is the cross-sectional area of the flow in the swirl element, and $V_{m}$ is the mixture velocity in the pipe.
  Considering a drift-flux model, the velocity of the gas in the flow is given by a term proportional to the mixture velocity plus a slip velocity:
  (A3) $$u_{g}=CV_{m}+V_{slip},$$
  where *C* and $V_{slip}$ are constants, which depend on the flow pattern.
  Figure A1 shows the relation obtained between gas velocity, detected by the double-layer wire-mesh sensor, and the mixture velocity, calculated based on the flow rates of liquid and gas at the location of the wire-mesh, for the points of Appendix A, in which slug flow is observed in the experiments. A least-squares fit of the graph based on the drift-flux model ($V_{wms}=CV_{m}+V_{slip}$) leads to C=1.34 and $V_{slip}=0.43$ m/s.
  The flow patterns inside the vanes of the swirl element are unknown, and it is assumed that the upstream flow patterns (typically slug) are maintained during the passage of the flow through the swirl element, which allows computing the gas velocity based on the same drift-flux coefficients of the upstream flow:
  (A4) $$u_{g,se}=CV_{m,se}+V_{slip},$$
  with C=1.34 and $V_{slip}=0.43$ m/s when the slug flow is observed upstream of the swirl element.
  Instead of using $V_{slip}$, which is flow pattern dependent, the expression can be manipulated to include the wire-mesh sensor velocity explicitly, making it independent of the flow pattern (as long as the hypothesis of the maintenance of the upstream flow pattern along the vanes of the swirl element is still valid):
  (A5) $$u_{g,se}=C\left(\frac{A}{A_{se}}-1\right)V_{m}+V_{wms},$$
  leading to $u_{g,se}=1.13\;V_{m}+V_{wms}$ for the experimental facility (the area ratio is equal to 1.84 from the geometry of the swirl element). The delay across the swirl element is given by its length divided by the gas velocity, i.e., $\tau_{2}=3.6\;D/u_{g,se}$.
iii. The gas moves at speed $u_{g}=q_{g}/A_{g}$ between the swirl element outlet and the camera, where $A_{g}$ is the area of the gas core measured by the camera ($A_{g}=\alpha A$). Therefore, $\tau_{3}=5.71\;DA\alpha /q_{g}$.

Figure A2 shows the three components of the total delay as a function of the wire-mesh sensor velocity for each experimental point of Appendix A. The figure shows that the region between the wire-mesh sensor and the swirl element contributes to around 60% of the total delay between the two signals, followed by the swirl element with around 30% and the region inside the inline separator with only around 10% of the total delay. The small contribution of the gas core section in the total delay is expected, since the area of the core is small and the phase moves much faster inside the swirl separator than in the regions upstream.

Combining the individual delays, the total delay between the wire-mesh and the camera can be estimated from conservation of mass as

(A6) $$\tau =\frac{4.4D}{V_{wms}}+\frac{3.6D}{1.13V_{m}+V_{wms}}+\frac{5.71DA\alpha }{q_{g}}$$

It is worth mentioning that, although Equation (A6) leads to a physical understanding of the delay and a good match with the experiments, the fit of Figure 18 is more convenient to use in predictions of the core based on the WMS signal since it only depends on data acquired by the sensor, while Equation (A6) depends on flow rates and core properties that are not measured by the technique.
